# The Natural Historian's Guide to the CT Galaxy: Step-by-Step Instructions for Preparing and Analyzing Computed Tomographic (CT) Data Using Cross-Platform, Open Access Software

**DOI:** 10.1093/iob/obaa009

**Published:** 2020-04-10

**Authors:** T J Buser, O F Boyd, Á Cortés, C M Donatelli, M A Kolmann, J L Luparell, J A Pfeiffenberger, B L Sidlauskas, A P Summers

**Affiliations:** 1 Department of Fisheries and Wildlife, Oregon State University, Corvallis, OR, USA; 2 Department of Integrative Biology, Oregon State University, Corvallis, OR, USA; 3 Department of Biology, University of Ottawa, Ottawa, ON, USA; 4 Department of Biological Sciences, George Washington University, Washington, DC, USA; 5 Department of Biology, Temple University, Philadelphia, PA, USA; 6 Department of Biology and SAFS, University of Washington, Friday Harbor Laboratories, Friday Harbor, Washington, DC, USA

## Abstract

The decreasing cost of acquiring computed tomographic (CT) data has fueled a global effort to digitize the anatomy of museum specimens. This effort has produced a wealth of open access digital three-dimensional (3D) models of anatomy available to anyone with access to the Internet. The potential applications of these data are broad, ranging from 3D printing for purely educational purposes to the development of highly advanced biomechanical models of anatomical structures. However, while virtually anyone can access these digital data, relatively few have the training to easily derive a desirable product (e.g., a 3D visualization of an anatomical structure) from them. Here, we present a workflow based on free, open source, cross-platform software for processing CT data. We provide step-by-step instructions that start with acquiring CT data from a new reconstruction or an open access repository, and progress through visualizing, measuring, landmarking, and constructing digital 3D models of anatomical structures. We also include instructions for digital dissection, data reduction, and exporting data for use in downstream applications such as 3D printing. Finally, we provide Supplementary Videos and workflows that demonstrate how the workflow facilitates five specific applications: measuring functional traits associated with feeding, digitally isolating anatomical structures, isolating regions of interest using semi-automated segmentation, collecting data with simple visual tools, and reducing file size and converting file type of a 3D model.

## Synopsis

**(ARABIC) ** عربى دليل المؤرخ الطبيعي لمجرة التصوير المقطعي المحوسب CT: تعليمات خطوة بخطوة لإعداد وتحليل بيانات التصوير المقطعي المحوسب (CT) باستخدام برامج مفتوحة متداخلة المنصاتإعداد: ثاديوس بوسر ^1 ^، أوليفيا بويد ^2 ^، ألفارو كورتيز ^1 ^، كاساندرا دوناتيللي ^3 ^، ماثيو كولمان ^4 ^، جينيفير لوباريل ^1 ^، جان فايفنبرغر ^5 ^، بريان سيدلوكاس ^1 ^، آدم سامرز ^6 ^نبذة مختصرةأدى انخفاض تكلفة الحصول على بيانات التصوير المقطعي المحوسب (CT) إلى زيادة الجهد العالمي لحوسبة تشريح عينات المتاحف. أنتج هذا الجهد ثروة من نماذج التشريح ثلاثية الأبعاد رقمية مفتوحة متاحة لأي شخص من خلال شبكة الإنترنت. التطبيقات المحتملة لهذه البيانات واسعة النطاق، وتتراوح من طباعة نماذج ثلاثية الأبعاد لأغراض تعليمية بحتة إلى تطوير نماذج ميكانيكية حيوية متقدمة للغاية للتركيبة البنيوية للهياكل. ومع ذلك، وبالرغم من إمكانية أي شخص الوصول إلى هذه البيانات الرقمية، إلا أن نسبة ضئيلة من المستخدمين حصلت على التدريب اللازم لاستخلاص منتج مرغوب فيه بسهولة (مثلا تصور ثلاثي الأبعاد لبنية تشريحية). نقدم هنا مسار عملي مبني على برنامج مجاني مفتوح المصدر ومتعدد المنصات لمعالجة بيانات الأشعة المقطعية. ونقدم تعليمات تفصيلية خطوة بخطوة تبدأ بالحصول على بيانات التصوير المقطعي المحوسب من إعمار تركيبة جديدة (new reconstruction) أو من حافظة بيانات مفتوحة، والتقدم إلى التصور والقياس وتحديد المعالم وإنشاء نماذج ثلاثية الأبعاد رقمية للهياكل التشريحية. ونشمل أيضًا تعليمات حول التشريح المحوسب وتقليص البيانات وتصديرها للاستخدام في تطبيقات نهائية مثل الطباعة ثلاثية الأبعاد. أخيرًا، نقدم مقاطع فيديو تكميلية ومسارات عمل لتوضيح كيف تبسّط الإجراءات خمس استخدامات محددة: قياس السمات الوظيفية المرتبطة بالتغذية، وعزل الهياكل التشريحية رقميًا، وعزل المناطق ذات الاهتمام باستخدام التقطيع شبه الآلي، وجمع البيانات باستخدام أدوات بصرية بسيطة، وتقليص حجم الملف وتحويله لنموذج ثلاثي الأبعاد.

**ITALIANO (ITALIAN)** **La guida dello storico naturale alla galassia CT: istruzioni passo-passo per preparare e analizzare i dati tomografici calcolati (CT) utilizzando un software multipiattaforma ad accesso aperto** **Thaddaeus J. Buser, Olivia F. Boyd, Álvaro Cortés, Cassandra M. Donatelli, Matthew A. Kolmann, Jennifer L. Luparell, Janne A. Pfeiffenberger, Brian L. Sidlauskas, Adam P. Summers** **ASTRATTO**La decrescita nel costo dell'acquisizione di immagini attraverso la tomografia computerizzata (TC) ha incentivato uno sforzo globale nella digitalizzazione anatomica di campioni museali. Tali sforzi sono risultati in un'abbondanza di modelli anatomici 3 D accessibili al pubblico su internet in formato open access. I potenziali impeghi di questi dati sono molteplici, spaziando dalla stampa 3 D per scopi puramente educativi allo sviluppo di modelli biomeccanici avanzati di strutture anatomiche. Ciononostante, seppure questi dati siano in teoria accessibili a chiunque, relativamente poche persone hanno la preparazione necessaria per derivarne un prodotto fruibile, come per esempio una visualizzazione 3 D di una struttura anatomica. In questo lavoro presentiamo un workflow basato su un software open access gratuito e multipiattaforma che permette l'elaborazione di dati acquisiti attraverso tomografia computerizzata. Inoltre, vengono fornite istruzioni passo-per-passo cominciando dall'acquisizione di dati tomografici a partire da una nuova ricostruzione o da un archivio open access e proseguendo con la visualizzazione, misurazione, mappatura ed infine costruzione di modelli 3 D di strutture anatomiche. In aggiunta, includiamo istruzioni per la dissezione digitale nonché per la riduzione e l'esportazione dei dati per usi successivi, come per esempio la stampa 3 D. Infine offriamo video e procedure supplementari nei quali viene dimostrato come tale workflow assista in cinque diverse applicazioni: la misurazioni di tratti funzionali associati all'alimentazione, l'isolamento digitale di strutture anatomiche, l'isolamento di regioni di interesse usando la segmentazione semi-automatizzata, l'acquisizione di dati attraverso semplici mezzi visuali, la riduzione delle dimensioni dei file e la conversione al formato 3 D.

**РУССКИЙ (RUSSIAN)** **Руководство естествоиспытателя по миру КТ: пошаговые инструкции для подготовки и анализа данных компьютерной томографии (КТ) с использованием кроссплатформенного программного обеспечения с открытым доступом** **Таддеус Дж. Бусер, Оливия Ф. Бойд, Альваро Кортес, Кассандра М. Донателли, Мэтью А. Колманн, Дженнифер Л. Лупарелл, Янн А. Пфейффенбергер, Брайан Л. Сидлаускас, Адам П. Саммерс** **АБСТРАКТ**Снижение стоимости получения данных компьютерной томографии (КТ) привело к массовой оцифровке музейных экспонатов, что позволило создать множество цифровых анатомических 3 D-моделей. Эти модели находятся доступны любому с помощью сети Интернет. Потенциальные возможности применения этих цифровых моделей широки и включают в себя печать 3 D-макетов для образовательных целей и разработку высокотехнологичных биомеханических анатомических моделей. Несмотря на то, что практически каждый желающий имеет открытый доступ к этим цифровым моделям, преобразовать их в желаемый продукт (например, трехмерную визуализацию анатомической структуры) могут относительно немногие. В данной статье мы описываем рабочий процесс, основанный на использовании бесплатного кроссплатформенного программного обеспечения с открытым исходным кодом для обработки данных КТ. Мы предлагаем пошаговые инструкции, начиная с получения данных КТ из новой реконструкции или хранилища с открытым доступом. Затем, мы описываем возможности визуализации, измерения, ориентирования и создания цифровых трехмерных моделей анатомических структур. Кроме того, мы включили инструкции по цифровому анализу, сокращению данных и экспорту данных для использования в последующих процессах, таких как 3 D-печать. В конце мы предоставили дополнительные видео и описания решения пяти конкретных задач: измерение функциональных признаков, связанных с кормлением, цифровая извлечение анатомических структур, излечение интересующих пользователя областей с помощью полуавтоматической сегментации, сбор данных с помощью простых визуальных инструментов и сокращение размера файлов и преобразование различных типов файлов в 3 D-модели.

**DEUTSCHE (GERMAN)** **Per Anhalter durch die CT Galaxie: Arbeitsablauf für die Verarbeitung und Analyse von Computertomographiedaten (CT) mit plattformübergreifenden, Open Source Software** **Thaddaeus J. Buser, Olivia F. Boyd, Álvaro Cortés, Cassandra M. Donatelli, Matthew A. Kolmann, Jennifer L. Luparell, Janne A. Pfeiffenberger, Brian L. Sidlauskas, Adam P. Summers** **ABSTRAKT**Die Kostensenkung für die Erhebung von Computertomographiedaten (CT) haben die weltweiten Bemühungen zur Digitalisierung der Anatomie von Museumsexemplaren vorangetrieben. Eine Fülle von digitalen 3 D-Modellen der Anatomie sind durch diese Bemühungen erzeugt worden, die allen mit Internetzugang offen zur Verfügung stehen. Die Anwendungsmöglichkeiten dieser Daten sind breit gefächert und reichen von gedruckten 3 D Modellen für rein pädagogische Zwecke bis zur Entwicklung hochentwickelter biomechanischer Modelle von anatomischen Strukturen. Während jeder Zugriff auf diese digitalen Daten hat, haben relativ wenige die Ausbildung aus ihnen ein wünschenswertes Produkt (z. B. eine 3 D-Visualisierung einer anatomischen Struktur) zu kreieren. Hier präsentieren wir einen Arbeitsablauf, der auf einer freien Open Source, plattformübergreifenden Software zur Verarbeitung von CT-Daten basiert. Wir bieten Schritt-für-Schritt-Anleitungen, die mit der Erhebung von CT-Daten aus einer neuen Rekonstruktion oder einem Open-Access-Repository beginnen, und schreiten fort mit der Visualisierung, Messung, Platzierung von landmarks und den Aufbau digitaler 3 D-Modelle anatomischer Strukturen. Weitere Anweisungen beinhalten unter anderem virtuelle Sezierung, Datenreduktion, und den Export von Daten für die Nutzung in nachgelagerten Anwendungen (z. B. 3 D-Drucken). Schließlich bieten wir zusätzliche Videos und Arbeitsabläufe an, die demonstrieren, wie der Arbeitsablauf fünf spezifische Anwendungen ermöglicht: die Messung funktionaler Merkmale im Zusammenhang mit Essen, die digitale Isolierung anatomischer Strukturen, die Isolierung von Interessengebieten mit halbautomatischer Segmentierung, das Sammeln von Daten mit einfachen visuellen Tools, die Reduzierung der Dateigröße und das Konvertieren des Dateityps eines 3 D-Modells.

**ČEŠTINA (CZECH)** **Průvodce po stopách CT galaxii: krok za krokem pokyny pro přípravu a analýzu vypočtených tomografických (CT) dat pomocí multiplatformového softwaru s otevřeným přístupem** **Thaddaeus J. Buser, Olivia F. Boyd, Álvaro Cortés, Cassandra M. Donatelli, Matthew A. Kolmann, Jennifer L. Luparell, Janne A. Pfeiffenberger, Brian L. Sidlauskas, Adam P. Summers** **ABSTRAKTNÍ**Snížení nákladů na pořízení dat z výpočetních tomografií (CT) vyvolalo celosvětové úsilí o digitalizaci anatomických muzejních vzorků. Vznikla velká sbírka dat anatomických exemplařů a digitálních 3 D modelů, která je každému přístupná přes internet. Možné aplikace těchto dat jsou široké, od 3 D tisku pro čistě vzdělávací účely až po vývoj vysoce pokročilých biomechanických modelů anatomických struktur. Přestože k těmto digitálním datům může přistupovat prakticky kdokoli, relativně málo z nich má védomosti, jak získat požadovaný produkt (např. 3 D vizualizaci anatomické struktury). Zde představujeme pracovní postup založený na bezplatném otevřeném zdrojovém softwaru napříč platformami pro zpracování dat CT. Poskytujeme postupné pokyny, které začínají získáváním CT dat z nové rekonstrukce nebo úložiště s otevřeným přístupem a postupují vizualizací, měřením, orientací a konstrukcí digitálních 3 D modelů anatomických struktur. Zahrnujeme také pokyny pro digitální disekci, redukci dat a export dat pro použití v navazujících aplikacích, jako je 3 D tisk. Nakonec poskytujeme doplňková videa a pracovní postupy, které demonstrují, jak pracovní postup usnadňuje pět specifických aplikací: měření funkčních znaků spojených s krmením, měření digitálně izolační anatomické struktury, oddéleni zájmových oblastí pomocí poloautomatizované segmentace, sběr dat pomocí jednoduchých vizuálních nástrojů a zmenšení souboru dat a zmény formátu do 3 D modelů.

**PORTUGUÊS (PORTUGUESE)** **O Guia da Galáxia da Tomografia Computadorizada para um Biólogo: instruções passo a passo para preparar e analisar dados tomográficos usando um software gratuito de acesso aberto** **Thaddaeus J. Buser, Olivia F. Boyd, Álvaro Cortés, Cassandra M. Donatelli, Matthew A. Kolmann, Jennifer L. Luparell, Janne A. Pfeiffenberger, Brian L. Sidlauskas, Adam P. Summers** **RESUMO**O custo decrescente da obtenção de dados de Tomografia Computadorizada (TC) alimentou um esforço global para digitalizar espécimes depositados em museus. Esse esforço produziu uma grande variedade de modelos digitais 3 D com dados de anatomia, disponíveis para qualquer pessoa com acesso à Internet. As aplicações potenciais desses dados são amplas, desde a impressão 3 D para fins puramente educacionais, até o desenvolvimento de modelos biomecânicos de estruturas anatômicas altamente avançados. No entanto, enquanto praticamente qualquer pessoa pode acessar esses dados digitais, relativamente poucos têm o treinamento para obter facilmente um produto de interesse (por exemplo, uma visualização 3 D de uma estrutura anatômica). Aqui, apresentamos um tutorial baseado em um software gratuito de código aberto e multiplataforma para o processamento de dados de TC. Fornecemos instruções passo a passo que começam com a obtenção de dados de TC a partir de uma nova reconstrução ou num repositório de acesso aberto, e progredimos através da visualização, medição, marca de referência e construção de modelos digitais 3 D de estruturas anatômicas. Também incluímos instruções para dissecação digital, redução de dados e exportação de dados para uso em aplicativos posteriores, como os de impressoras 3 D. Por fim, fornecemos vídeos e tutoriais suplementares que demonstram como o tutorial facilita cinco aplicações específicas: medir características funcionais associadas à alimentação, isolar estruturas anatômicas digitalmente, isolar regiões de interesse usando segmentação semi-automática, coletar dados com ferramentas visuais simples, e reduzir o tamanho de arquivo e converter o tipo de arquivo do modelo 3 D.

**FRANÇAIS (FRENCH)** **Guide de l'historien de la nature à travers la galaxie TDM: instructions étape par étape pour la préparation et l'analyse de données tomodensitométrique** (**TDM**) **à l'aide d'un logiciel à accès ouvert multiplateformeThaddaeus J. Buser, Olivia F. Boyd, Álvaro Cortés, Cassandra M. Donatelli, Matthew A. Kolmann, Jennifer L. Luparell, Janne A. Pfeiffenberger, Brian L. Sidlauskas, Adam P. SummersRÉSUMÉ**Le coût décroissant de l'acquisition de données tomodensitométriques (TDM) a alimenté un effort mondial pour numériser l'anatomie des spécimens de musée. Cet effort a produit une multitude de modèles d'anatomie numérique 3 D en accès libre accessibles à tous ceux qui ont accès à Internet. Les applications potentielles de ces données sont vastes, allant de l'impression 3 D à des fins purement pédagogiques au développement de modèles biomécaniques de structures anatomiques très avancés. Cependant, alors que pratiquement tout le monde peut accéder à ces données numériques, relativement peu ont la formation nécessaire pour en tirer facilement un produit intéressant (par exemple, une visualisation 3 D d'une structure anatomique). Ici, nous présentons un flux de travail basé sur un logiciel gratuit, à accès ouvert et multiplateforme pour le traitement des données TDM. Nous fournissons des instructions étape par étape qui commencent par l'acquisition de données TDM à partir d'une nouvelle reconstruction ou d'un référentiel en accès gratuit, et progressent à travers la visualisation, la mesure, le marquage et la construction de modèles numériques 3 D de structures anatomiques. Nous incluons également des instructions pour la dissection numérique, la réduction des données et l'exportation de données à utiliser dans des applications en aval telles que l'impression 3 D. Enfin, nous proposons des vidéos et des workflows supplémentaires qui montrent comment le workflow facilite cinq applications spécifiques: mesurer les traits fonctionnels associés à l'alimentation, isoler numériquement les structures anatomiques, isoler les régions d'intérêt à l'aide de la segmentation semi-automatisée, collecter des données avec des outils visuels simples, réduire la taille du fichier et convertir le type de fichierd'un modèle 3 D.

## Introduction

The applications of three-dimensional (3D) visualizations of internal anatomy are varied and vast, spanning a galaxy of analytical possibilities. Recently, the increased ease of gathering such data has led to their widespread adoption in the comparative morphological community. The embrace of this new data type has, in turn, catalyzed many recent biological discoveries, such as revealing brain and muscle activity during bird flight (positron emission tomography scanning; Gold et al. 2016), determining how blood circulates through vasculature (magnetic resonance imaging; O'Brien and Williams 2014; O'Brien 2017), revealing the function of the appendicular skeleton during locomotion and feeding in live sharks (X-ray Reconstruction of Moving Morphology, 3D fluoroscopy coupled with computed tomographic [CT] animation; Camp et al. 2017; Scott et al. 2019) and reconstructing the feeding behavior of long-extinct monsters of the deep (CT imaging of *Helicoprion*; Tapanila et al. 2013). Other researchers have used 3D digitization to educate and inform. Anatomical models of living and extinct taxa can be built digitally so that students can manipulate, dissect, and scale anatomical structures online (see Rahman et al. 2012; Manzano et al. 2015), used to make 3D prints of missing bones of incomplete physical specimens, or print whole rare or otherwise difficult to acquire specimens for use in teaching comparative anatomy (Gidmark 2019; Staab 2019). For example, the anatomically accurate, 3D printed, vertebrate skull magnetic puzzles by Singh et al. (2019) allow students to understand how different parts of the skull fit together. 

Open source efforts like MorphoSource (Boyer et al. 2016; morphosource.org) and DigiMorph (digimorph.org) aggregate thousands of digital 3D models into anatomical libraries and serve them freely to researchers, teachers, and laypersons alike. Like other synthetic, open access approaches to data management and data sharing (Sidlauskas et al. 2009; Whitlock 2011; but see also Hipsley and Sherratt 2019), these repositories encourage data reuse, reanalysis, and reinterpretation, and have ushered in a digital renaissance of comparative morphology.

Most of the 3D images in the online digital libraries result from computed tomography scanning, commonly known as “CT” or “cat” scanning, which benefits from the quadruple advantages of non-destructivity, shareability, printability, and affordability (Cunningham et al. 2014; Sutton et al. 2014). CT scanning neither invades, modifies, or destroys the original sample. The digital nature of CT data makes it easy to share via open-access platforms and has sparked “big data” initiatives, such as oVert (floridamuseum.ufl.edu/overt) and the #ScanAllFishes projects (adamsummers.org/scanallfish). The simplicity of converting CT scans to digital “surfaces” allows almost any anatomical structure to be 3D printed, even permitting structures to be artificially warped, scaled, or mirrored to fit experimental or teaching needs (Stayton 2009). Scans can also be converted into digital “meshes” which can be used to gather 3D geometric morphometrics data (Lawing and Polly 2010), model the reaction forces on the structures using finite element analysis (FEA; see Hulsey et al. 2008), predict fluid flow around structures using computational fluid dynamics (see Inthavong et al. 2009), study multibody dynamics (Lautenschlager et al. 2016), or render and animate 3D objects (Garwood and Dunlop 2014).

Perhaps most importantly, the decreasing cost, size, and complexity of CT hardware, and the development of open source software like Horos (https://horosproject.org/) or 3D Slicer (Fedorov et al. 2012; Kikinis et al. 2014, https://download.slicer.org) has opened access to scientists working outside the biomedical arena. Aspiring digital anatomists no longer need to seek time on the multi-million-dollar, room-sized set-ups in hospitals, but can use desktop machines costing far less. The spread of these smaller systems, often purchased through collaborative interdepartmental funding opportunities, has drastically decreased the cost per study, increased the willingness of researchers to share their data, and caused CT data to explode in popularity, even among scientists who lack access to CT hardware (see Davies et al. 2017). The methods have now transcended biomedical and anthropological research to penetrate fields like organismal taxonomy, paleontology, comparative anatomy, and physiology, as well as biomechanics and biomimetics (Cohen et al. 2018; Divi et al. 2018; Santana 2018; Rutledge et al. 2019). The advantages of biodiversity and taxonomical research cannot be understated as rare, endemic, and understudied taxa can now be shared widely. More open access to specimens allows for systematic hypotheses to be updated, re-examined, and replicated, and each scan preserves an in silico virtual record of morphology for posterity. Metaphorically, each of these virtual specimens can be considered a point of light in a vast and growing constellation depicting the world's biological diversity. Those researchers able to navigate that starfield, which we dub the CT galaxy, will be poised to visualize and analyze biodiversity in ways never before possible.

As is typical when technologies become newly affordable and accessible, the pace of method development has far outstripped the pace of training. Though many researchers and educators have become aware of CT's potential, relatively few have been able to participate in focused training workshops. Strides have been made in establishing best practices in the process of CT scanning itself, and in the curation of 3D data (Davies et al. 2017; Keklikoglou et al. 2019). However, the only available training protocols for analyzing the CT data after they have been gathered have been *ad hoc* efforts developed within research groups and passed among scientists via email and similar channels. This contribution aims to democratize access to such training by publishing an open-access workflow using freely available and cross-platform software.

Herein, we outline a set of practices in the production, visualization, and analysis of CT data. We have found this workflow saves time and money while maximizing efficiency. We hope that these suggestions tempt the uninitiated to experiment with CT methods for the first time or ease the struggle of learning new techniques. To that end, we focus on those often-tedious nuances of data preparation, formatting, and navigating software that commonly hinder progress in CT-based studies of anatomy, functional morphology, and macroevolution. We also emphasize tools useful for creating pedagogical aids such as 3D prints and images of anatomical structures. Whenever possible, we include steps for data-reduction that help to make processing time more reasonable for older/slower machines, although most any reasonably up-to-date machines (e.g., Mac OS X Lion 10.7.3, Windows 7, Ubuntu 10.10, or newer) can perform all manipulations and analyses herein.

## Software

This workflow is designed to be completely open to any researcher, educator, or enthusiast. Generally speaking, the only limitation is access to a computer with at least 8 GB of random access memory (RAM), though this depends mostly on the size of the file to be analyzed. For optimal performance, we recommend that the data file not exceed one-tenth to one-fourth the size of the available RAM on your computer. For example, if you have 8 GB of RAM, your data file should be no larger than 0.8 GB (800 MB) to 2 GB. If the file that you intend to analyze is larger than this range, we include a variety of steps below for down-sampling or working around the computationally and/or memory-intensive steps of CT analysis. There are a variety of software programs available to process CT data, and the programs we employ are freely available and cross-platform (Table 1; see also Abel et al. 2012). Before beginning the workflow, ensure that you have installed the latest stable version of the ImageJ (Schneider et al. 2012; Rueden et al. 2017) expansion Fiji (Schindelin et al. 2012, https://fiji.sc; we use ImageJ v2.0.0 herein) and 3D Slicer (https://download.slicer.org; v.4.10.2 used herein). We also recommend that users interested in working with 3D surface meshes install MeshLab (Cignoni et al. 2008; Pietroni et al. 2010, www.meshlab.net; v. 2016.12 used herein). If your computer has a dedicated graphics card, you can use it in Slicer to reduce lag time when rendering your data in 3D. The process for telling Slicer to use your graphics card will vary based on your machine, operating system, and the brand of card. Generally, there will be an option in the automatically installed graphics card software (NVIDIA—NVIDIA Control Panel, AMD—AMD Catalyst^™^ Control Center, etc.) to select which programs you want to use the card by default. Set this up before running Slicer (you will likely have to re-start your machine). Alternatively, users can manually designate the graphics card within Slicer in the volume rendering step (see Step 7.a.ii.1, below), but this action must be repeated in every session. Finally, ensure that there is adequate hard drive space on your machine for storing the CT dataset and derivative products thereof. Approximately 10 GB will be adequate for all steps involved in this workflow using the example datasets. Users who wish to store and process several CT datasets should consider the size of the datasets with respect to their available hard drive space. Datasets available on MorphoSource range from ∼200 MB to 10 GB in size, and we recommend a storage capacity of several terabytes (TB) for users wishing to engage in extensive (i.e., high sample size) studies using CT data.

**Table 1:** Open-source, cross-platform software for visualizing and analyzing CT data

Software	URL	Operating system(s)	Recommended uses; advantages	Limitations
Drishti	https://github.com/nci/drishti	All (Windows, Mac OS, Linux)	Tools for image viewing, editing, processing, surface and volume rendering, mesh generation, animation; intuitive user interface	Computationally demanding for volume rendering
SPIERS	https://spiers-software.org/	All	Tools for slice registration, image viewing, editing, processing, surface rendering, mesh generation, animation; handles large datasets well even on older/slower machines	Three separate modules for aligning, editing, and viewing; only produces meshes
Blender	https://www.blender.org/	All	Tools for editing 3D meshes, animation, video editing; intuitive user interface, customizable	Lacks tools for basic image processing (requires 3D model)
MeshLab	http://www.meshlab.net/	All	Tools for editing, analyzing, and refining 3D meshes	All processes restricted to working with meshes
3D Slicer	https://www.slicer.org/	All	Tools for image viewing, editing, processing, surface and volume rendering, file manipulation; intuitive user interface, extensible and customizable with a wide number of available modules, actively supported and developed	Works best on machines with faster graphics processing; may require downsampling of data
FIJI	https://fiji.sc/	All	Tools for image viewing, editing, processing, surface and volume rendering, file manipulation; extensible and customizable via the large number of purpose-built plugins available	Not the most intuitive interface for new users; some plugins no longer actively supported/developed
Biomedisa	https://biomedisa.de/	Not applicable (browser-based)	Semi-automated segmentation, in-browser viewer	Interpolates segments between labeled slices (no other image processing features)
MITK Workbench	http://mitk.org/	All	Tools for image viewing, editing, processing, surface and volume rendering, file manipulation, data management; customizable for developers	Interface may be challenging for new users
ITK-SNAP	http://www.itksnap.org/	All	Tools for manual and semi-automated segmentation; easily navigable user interface	Features limited to those related to segmentation
MANGO	http://ric.uthscsa.edu/mango/	All	Tools for image editing, processing, surface and volume rendering, file manipulation; command line accessible, customizable for developers	Interface may be challenging for new users

## Workflow

**Fig. 1:**
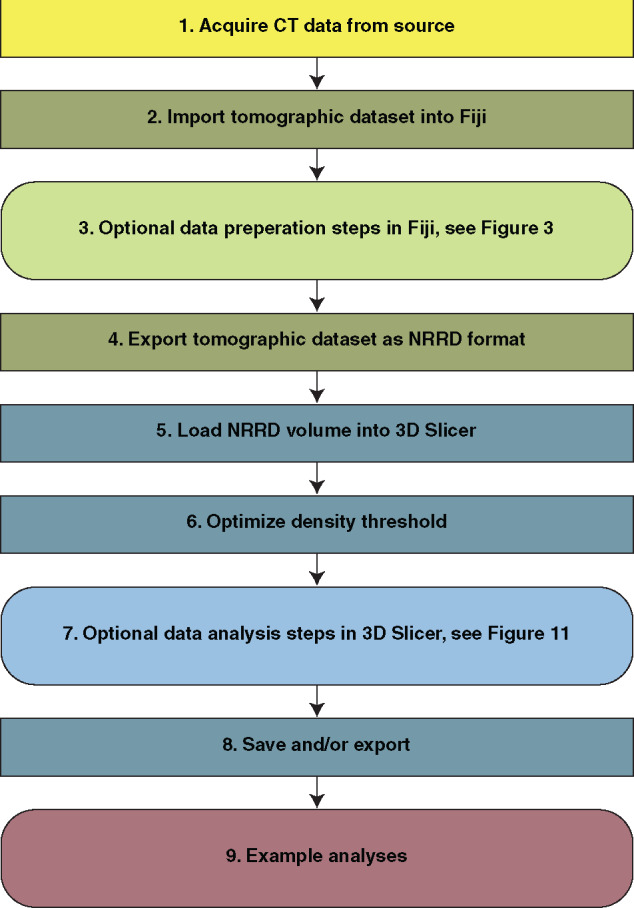
Flow chart of the steps involved in processing CT data described herein.

Figure 1 illustrates the steps of this workflow. Briefly, the user will acquire a tomographic dataset (Step 1) and read it into the program Fiji, where it can be manipulated to reduce file size before being exported as a single file in Nearly Raw Raster Data (NRRD) format (Steps 2–4). The user then imports the file into the program 3D Slicer, which can visualize the specimen(s) or region(s) of interest. Later steps demonstrate how to measure and landmark morphologies of interest, and/or export data for downstream applications (Steps 5–8). Step 7.f. specifically outlines the necessary workflow for generating the 3D surface renders for use in eventual 3D printing. The final step of the workflow (Step 9) presents five analytical examples to launch the reader's exploration of practical applications.

### Workflow steps

#### 1. Acquire CT data

**Fig. 2:**
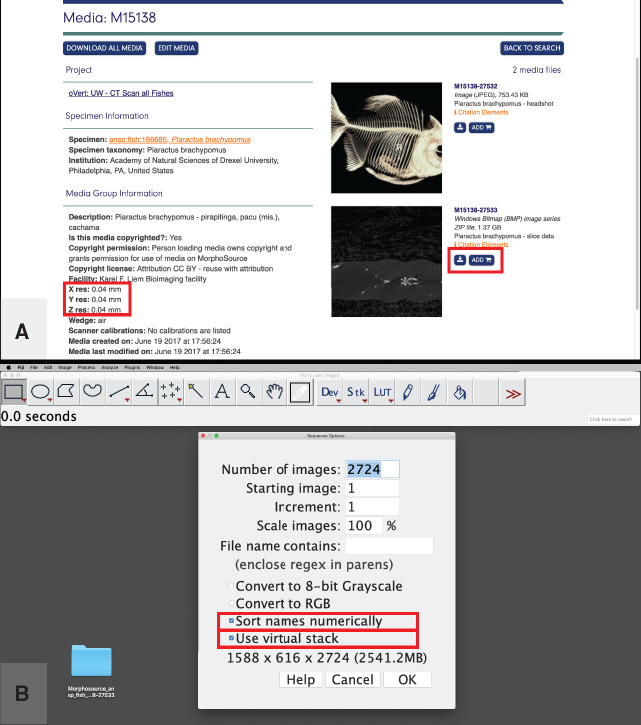
Acquiring CT data and loading them into the program Fiji. (**A**) The MorphoSource webpage (MorphoSource ID 15138) for a pacu (*P. brachypomus*) specimen from the Academy of Natural Sciences of Drexel University (specimen ID: Fish: 166685). The downloadable CT image stack (MorphoSource ID M15138-27533) and the specimen resolution data are each highlighted with a red box. (**B**) The image stack from (A) being imported into Fiji, with the recommended import options highlighted in red boxes. Illustrates Workflow Step 2.

Any CT reconstruction outputs a series of grayscale images that make up the CT “slices” of a specimen or specimens. The brightness of the pixels making up each image in the resulting tomographic series represents the X-ray attenuation of a given area within the scan, such that areas containing material with high X-ray attenuation (e.g., heavily-mineralized bone) appear white. In addition to the *x* and *y*-dimensions of the pixels, each slice contains a *z*-dimension (i.e., thickness) and thus each pixel actually represents a 3D volume of space, known as a voxel. The images in a tomographic series are usually in a standard format (e.g., .TIFF, .BMP, .JPEG, etc.) but they are often converted to a specialty format such as Digital Imaging and Communications in Medicine (DICOM). Whether your data come directly from CT reconstruction software or are downloaded from a CT data repository site such as MorphoSource.org (see Fig. 2A), OSF.io, or DigiMorph.org, you should move or copy the folder that contains the tomographic image series to a working location (we recommend a local file location such as the desktop rather than a remote drive). If the image series is in any format other than DICOM, locate the resolution/dimensionality data on either the data host website (Fig. 2A) or in the scanner log file. Note that MorphoSource removes the original scanner log file from their uploaded datasets, but the voxel dimensions can be found in the .CSV file accompanying your downloaded image stack dataset under the “x res,” “y res,” and “z res” columns. For the purposes of demonstrating the steps in our workflow, we will use a CT reconstruction of a pacu specimen (Pisces: Characiformes: *Piaractus brachypomus*; Academy of Natural Sciences of Drexel University, specimen ID: Fish: 166685), downloaded from MorphoSource.org (MorphoSource ID M15138-27533, see Fig. 2A and Supplementary Video S1). This is a modest-sized dataset (∼2.5 GB) that works well on most machines. However, readers whose machines have low available RAM (i.e., <8 GB) may experience lag times in processing this dataset, and we recommend instead that they follow along with a smaller dataset, such as the reconstruction of the sculpin *Porocottus allisi* (Pisces: Scorpaeniformes; University of Washington, Burke Museum of Natural History and Culture, specimen ID: UW 047873) available from MorphoSource.org (MorphoSource ID M15090-27349; file size: ∼193 MB). If you do not already have an account and login information for MorphoSource, you will need to create one to download these files. The time taken to download will vary with Internet connections and service providers.

#### 2. Import your tomographic stack of images into Fiji

While it is possible to import a tomographic image series directly into 3D Slicer, we have found that it is more reliable to first convert the image series into a single, NRRD format file. In fact, all of the optional tasks that are performed in Fiji in our workflow have analogs in 3D Slicer. Likewise, there are many tasks that we perform in 3D Slicer that could be performed in Fiji. However, we have found that the pairing of steps to the two programs outlined herein places each step in the program that performs it optimally. This minimizes instances of crashing and excessive wait times and thus maximizes the efficiency and robustness of the workflow. Familiarity with both Fiji and 3D Slicer greatly behooves the natural historian, especially once they become comfortable enough with the basic steps of CT image processing and begin to explore more advanced techniques.

- Open Fiji, go to “File,” then “Import,” and select “Image sequence.”
- Navigate to the folder containing your tomographic image stack and select the folder (Mac) or any image within the folder (Windows), and press “Open.”
- Next, Fiji will present you with a window of “Sequence Options,” where you can customize your import. If they are not checked already, check the box for “Sort names numerically” and “Use virtual stack.” Ensure that the “Increment:” is set to “1” and that the “Scale:” percent is set to “100.” Press “OK” (Fig. 2B).
  - Note: If desired, it is possible to reduce the file size of your stack through down-sampling, but do not attempt to do so here. See Step 3.e below.
  - Note: The use of the virtual stack reduces the time it takes to read-in the dataset, and we have found this helpful in saving time when cropping images. However, advanced users may wish to adjust parameters of the images (e.g., brightness and contrast) in Fiji. These steps are beyond the scope of this workflow, but for such users, we do not recommend using the virtual stack option, as this can introduce system errors when attempting to modify the image parameters of large datasets. For these advanced users, or users attempting to analyze datasets with file sizes larger than the available memory (RAM) on their computer, Supplementary Script S1 will enable FIJI to crop and/or adjust image parameters of image sequences with large file sizes.

#### 3. Optional steps—data preparation

**Fig. 3:**
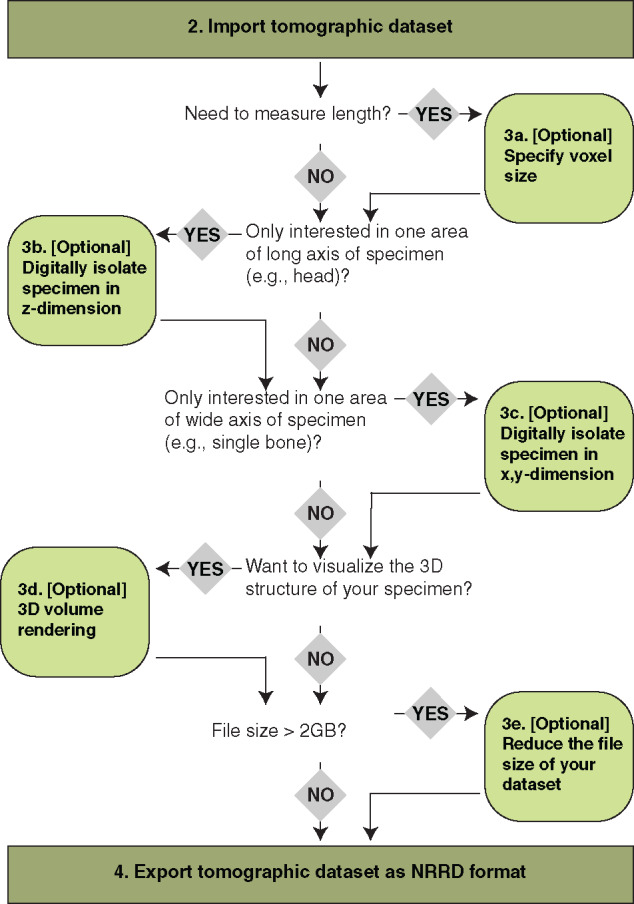
Decision tree for Workflow Steps 2–4, all performed in the using the program Fiji, which is an extension of the program ImageJ. Follow the decision tree to determine which options in Step 3 may be useful for your dataset and intended analyses.

There are several optional steps available within Fiji that serve to prepare the data for analysis in 3D Slicer. Use the decision tree illustrated in Fig. 3 to decide which (if any) optional steps are appropriate for your dataset and your intended analyses thereof.

**Fig. 4:**
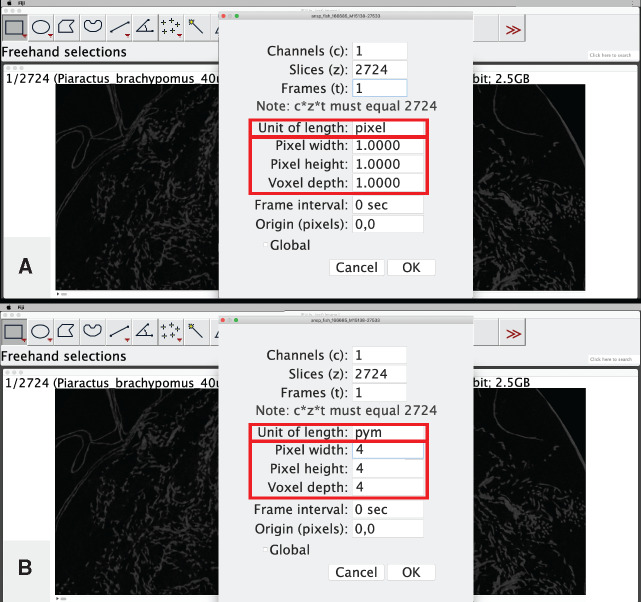
Specifying voxel size for the CT image stack from Workflow Step 1 using the program Fiji. The default dimensional data and unit of length (**A**) are replaced with the values indicated on the MorphoSource web page shown in Step 2.a that have been converted to units of “pym” (**B**). See text for details. Illustrates Workflow Step 3.a.
- **Digitally isolate your specimen/area of interest in the *z*-dimension:** For use when working with a large volume of data and/or when you are interested in only a portion of your CT dataset (e.g., you are interested in only skull but have scan data for the entire skeleton). This step helps reduce file sizes and increases processing speed.
  Locate the upper and lower bounds of your area of interest in the *z*-dimension by scrolling through the image stack using the scrub bar at the bottom of your image stack window (Fig. 5A).

- **Specify voxel size**: For use when any of your downstream analyses may include length. This step is highly recommended. Note: this step is usually not necessary if your tomographic dataset is in DICOM format.
  - Locate the *x*, *y*, and *z*-dimension length of your tomographic dataset. If your data come from the output of a CT reconstruction, the voxel/pixel size is indicated in the log file of the reconstruction (e.g., “Image Pixel Size (um)=39.989574”; in this case, it is implicit that this is the length of each dimension). If your data come from an online repository such as MorphoSource.org, this information may be indicated in the specimen data (Fig. 2A).
  - If necessary, convert the units so that a single number is present on the left side of the decimal place. For example, if the pixel size is reported as 39.989574 µm, convert it an arbitrary unit that represents ×10^−7^ m. For our example analysis, we will refer to this unit as a “pym,” and the pixel size of the above example would be 3.9989574 pym. The voxel dimensions for the pacu specimen are given as 0.04 mm (Fig. 2A), so would be represented as 4 pym. The pixel size of the sculpin reconstruction is “0.0299 mm” and we would represent this as 2.99 pym. This step is critical for avoiding arbitrary scaling and rounding issues in 3D Slicer, especially for users working without a dedicated graphics card in their machine.
  - In Fiji, click on the window that contains the image stack data that you opened in Step 2.
    1. Go to: “Image,” then select “Properties.”
    2. In the window that opens, change the “Unit of length” to whichever is most appropriate for your data (e.g., pym), and change the pixel/voxel dimensions to the appropriate dimension of your data. Press “OK” (Fig. 4).

**Fig. 5:**
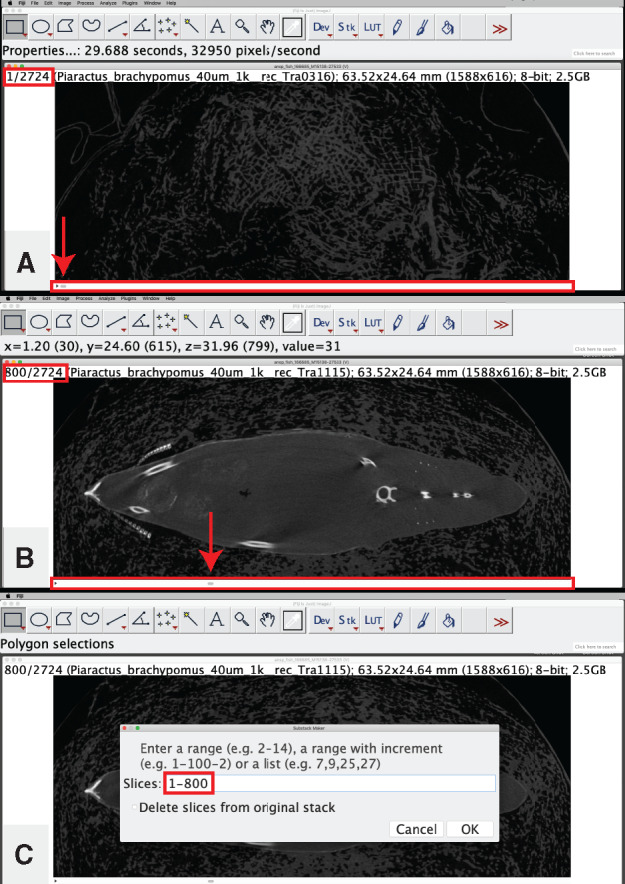
Digital isolation in the *z*-dimension on the image stack from Workflow Step 1. The scrub bar is highlighted with a red box in (**A** and **B**). The upper bounds of the region of interest is indicated on the scrub bar with a red arrow in (A), the lower bounds of the region of interest is indicated with a red arrow on the scrub bar in (B). The image number corresponding to the upper and lower bounds is highlighted with a red box in (A and B) (respectively). The image range containing the region of interest is specified in the “Slices:” range and highlighted with a red box in (C). Illustrates Workflow Step 3.b.
  Record the image number for each bound (Fig. 5A and B).
  Create a substack of just the images that contain your region of interest.
    1. Go to: “Image,” then “Stacks,” then “Tools,” and select “Make Substack…”
  In the “Substack Maker” window that pops up, input the range of images that contain your region of interest and press “OK” (Fig. 5C).
  The substack that you specified will open in a new stacks window titled “Substack” followed by the range that you specified in parentheses.
  Use this window for all additional steps.
    1. Note: It may help to close the original stack window to avoid confusion, though leaving it open is mostly harmless.
    2. Note: If attempting to analyze a dataset whose file size is larger than your available RAM, see Workflow Step 2.c.ii.
- **Digitally isolate your specimen/area of interest in the *x*, *y*-dimension**: For use when working with a large volume of data and you are interested in only a portion of your CT dataset (e.g., you are interested in only a single side of a bilaterally symmetric structure such as the cranium). This step may prove ineffective for highly 3D (e.g., coiled, spiraled) specimens, and user discretion is warranted in such instances.
  Select the “Rectangle” tool from the “(Fiji Is Just) ImageJ” toolbar (Fig. 6A).

**Fig. 6:**
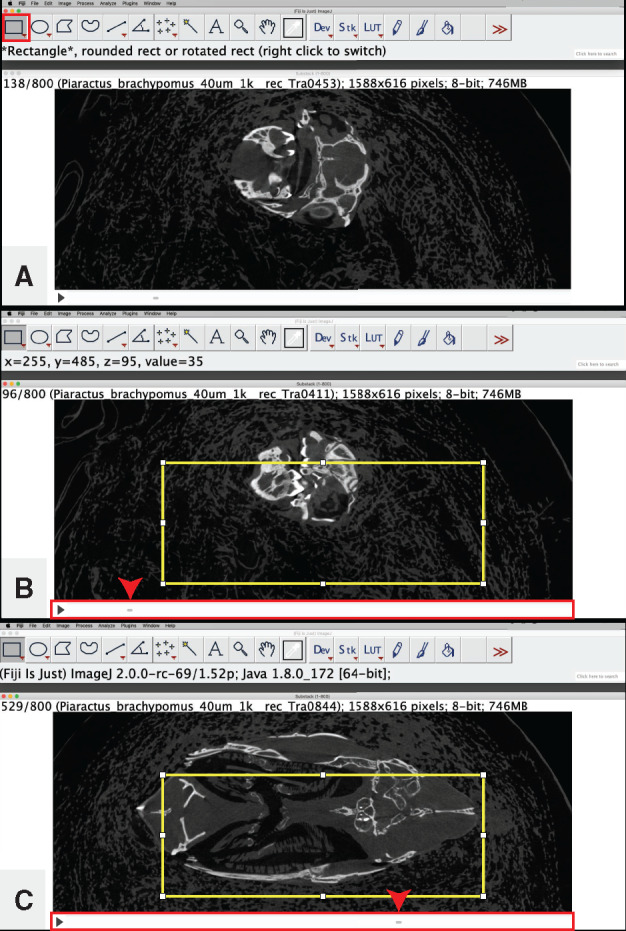
Digital isolation in the *x, y*-dimension of the image stack from Workflow Step 3.b. The rectangle tool (**A**) is used to encompass the region of interest (in yellow) (**B**, **C**). The scrub bar is highlighted in a red box and is used to locate the upper (B) and lower (C) bounds of the region of interest (denoted with red arrowhead). Illustrates Workflow Step 3.c.
  Use the rectangle tool to select an area of your scan that encompasses all of your specimen/area of interest.
    1. Use the rectangle tool on any image in your image sequence that contains your specimen (Fig. 6B).
    2. Use the scroll bar at the bottom of the window to visually check all images that contain your specimen to ensure that your highlighted region is not too large or too small (Fig. 6C).
      - Adjust borders of your rectangle as necessary.
  Crop the image stack to eliminate all the area outside of your rectangle.
    1. Go to “Image,” and select “Crop.”
    2. Note: If attempting to analyze a dataset whose file size is larger than your available RAM, see Workflow Step 2.c.ii.
- **Examine the 3D volume of your cropped image stack**: Use this step to visualize the 3D structure(s) contained within your image stack. This is useful for verifying that any previous digital dissection did not unintentionally remove any anatomical structures of interest. This step uses the “3D Viewer” plugin (Schmid et al. 2010), which comes pre-loaded in the Fiji software package.
  Go to “Plugins,” and select “3D Viewer.”
  Optional: Change the “Resampling factor:” from the default value of “2” to a higher number (e.g., ≥8) to decrease the amount of time it will take your computer to load the 3D volume rendering (Fig. 7A).

**Fig. 7:**
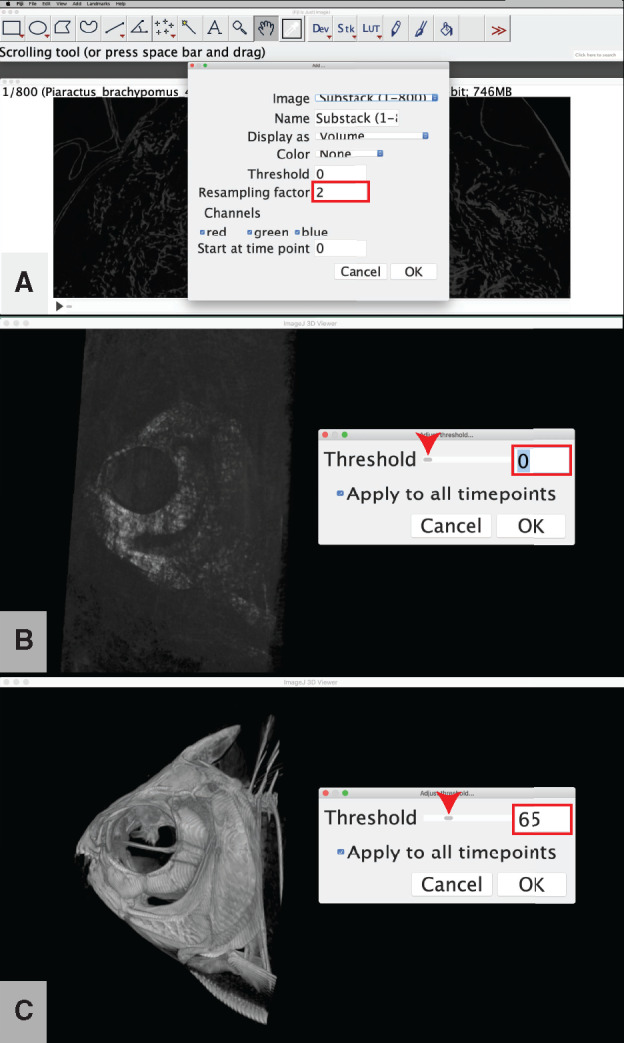
Visualization of CT data with a 3D volume rendering of the image stack from Workflow Step 3.c. The resampling factor (**A**) does not modify the underlying image data but decreases the resolution of the visualization in order to reduce loading time. We recommend a resampling factor of between 2 (small datasets and/or fast computer hardware) and 10 (large datasets and/or slow computer hardware). Adjust the threshold from its initial value (**B**) until the anatomy of interest is clearly visible (**C**). Illustrates Workflow Step 3.d.
    1. Note: This step will decrease the resolution of the rendering but will not affect the underlying slice data.
  Optional: Change the threshold value for the opacity of the volume rendering to highlight the denser materials (e.g., bone) in your scan.
    1. Select the “ImageJ 3D Viewer” window.
    2. Go to “Edit,” and select “Adjust threshold.”
    3. Slide the “Threshold:” scroll bar until the rendering highlights the material with the density of your choice (Fig. 7B and C).
    4. Press “OK.”
  Note: The volume rendering is a rotatable 3D area. Users with Microsoft Windows operating systems have reported issues with the rotation axis of the 3D volume in the ImageJ 3D Viewer. Until these issues are resolved by developers, Windows users can get around this issue by grabbing with the mouse within the ImageJ 3D Viewer window but outside of the 3D volume bounding box to rotate the area (i.e., click and drag in the black space surrounding the bounding box to properly rotate the area within the bounding box).
- **Reduce the file size of your image stack by down-sampling**: This step maintains the dimensionality of your specimen but reduces the resolution and thereby file size of the data. This can affect the visualization of minute structures on your specimen but may be necessary for downstream processing in programs that struggle with large file sizes (e.g., file sizes >2GB will crash 3D Slicer on most computers with ≤8GB of RAM). Advanced users working with large file sizes (or limited RAM) are encouraged to explore the program SPIERS (see Table 1), which can produce 3D models without loading the data into memory.
  The file size of your current image stack is indicated at the top of the stack window (Fig. 8A).

**Fig. 8:**
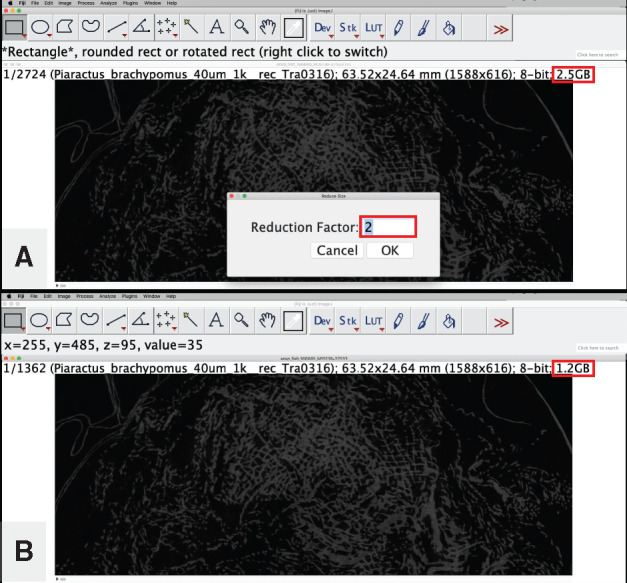
Reducing the file size of the dataset using a reduction factor applied to the image stack from Workflow Step 1. The initial file size and the reduction factor are each highlighted with a red box in (A). The resulting file size from applying the reduction factor is highlighted with a red box in (B). Illustrates Workflow Step 3.e.
  To reduce the size, go to “Image,” then “Stacks,” then “Tools,” and select “Reduce…”
  The default reduction factor is “2” (Fig. 8A), this reduces the number of slices and the size of your dataset by half and any single voxel in the new dataset will comprise the average value of a 2 × 2 × 2 cube of voxels in the original dataset.
    1. Tip: Simply divide the current file size of your dataset by the target file size to calculate the reduction factor. For example, if your current file size is 4.5 GB, and your target size is 1.5 GB, use a reduction factor of “3.”
  When the reduction process is complete, the new number of slices and the new file size will replace the old values at the top of the stack window (Fig. 8B).
  Note: if you did NOT set the dimensionality data for voxel size (i.e., Step 3.a above), your voxel dimensions will be given in “pixels” by default and you will need to manually change the voxel depth for your image stack after the reduction process is complete.
    1. Go to: Image → Properties.
      - Change “Voxel depth:” to whatever number you used as your reduction factor. For example, if you used a reduction factor of “3,” change the voxel depth to “3.”
      - Press “OK.”

#### 4. Export the image stack as NRRD format

- Go to “File,” then “Save As,” and select “Nrrd.”
- Specify file name and location and press “Save.”

#### 5. Load NRRD volume into 3D Slicer

**Fig. 9:**
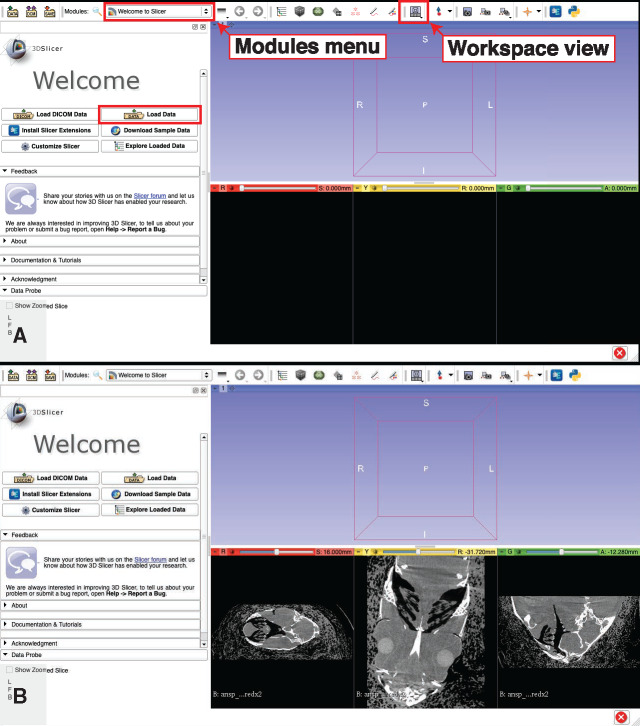
Loading image stack data into 3D Slicer. Use the drop-down menus to navigate the various modules and workspace views available in 3D Slicer (**A**). (**B**) The NRRD format tomographic dataset from Step 4 successfully loaded into 3D Slicer. Illustrates Workflow Step 5.
- Once the file has loaded, your screen should look something like Fig. 9B.
- If you specified the voxel size of your data (Step 3.a.), change the default unit of length in Slicer to match the units of your data.
  Click the “Edit” menu and select “Application Settings.”
    1. Select “Units” from the side menu.
    2. Check the box next to “Show advanced options”
    3. Under the “Length” submenu, change the “Suffix” from the default value of “mm” to the unit that you specified in Step 3.a. (e.g., for our data, we would set the suffix to “pym”).
      - Note: We recommend users also change the “Precision” level under the “Length” submenu from its default value of “3” to a value of 5–10. This will decrease errors and loss of information due to rounding.
    4. Note: Users with a dedicated graphics card can designate its use as a default setting here by selecting “Volume rendering” from the side menu, then changing the “Default rendering method:” to “VTK GPU Ray Casting,” changing the “Default quality” to “Normal,” and changing the “GPU memory size” to match the GPU memory on their machine.

3D Slicer is set up such that different sets of related tasks are grouped together in the “Modules:” drop-down menu. The program's default module is called “Welcome to Slicer” and this is where the program starts when it is first opened. In the “Welcome to Slicer” module, click on “Load Data” (Fig. 9A).

- Click on “Choose File(s) to Add.”
- Navigate to the NRRD file.
- Press “OK” and the NRRD will load.
- Ensure that the display is set to conventional:
  Click the “Workspace view” button to reveal a drop-down menu and select “Conventional” for optimal widescreen viewing (Fig. 9A).

#### 6. Optimize image contrast

This step adjusts the contrast of the image and is useful for any downstream step where visually differentiating structures are useful, such as trimming and editing segmentations (e.g., Step 7.c). However, it does not alter the underlying data; it simply alters how those data are visualized.

**Fig. 10:**
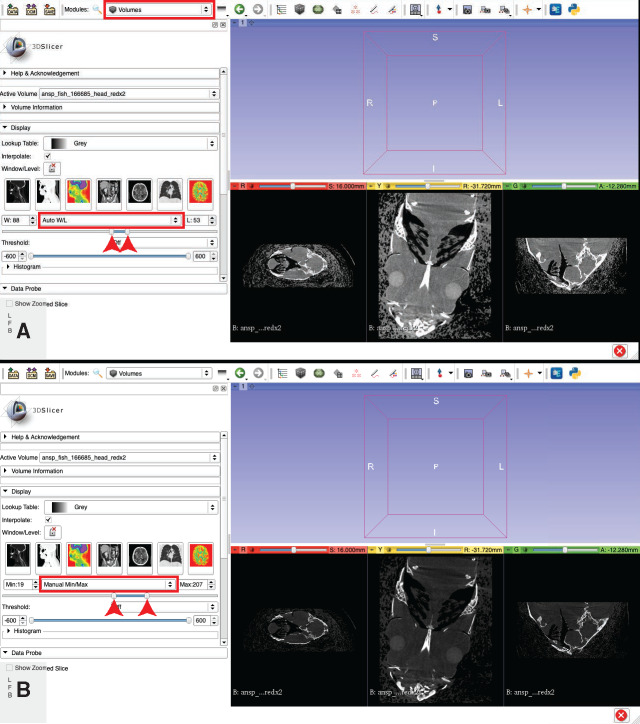
Image contrast optimization. The upper and lower bounds of the range of pixel values that will be displayed for the images in the tomographic stack are indicated with red arrowheads in the starting (default) values (**A**) and after manual optimization (**B**). Illustrates Workflow Step 6.
- Under the “Display” sub-menu there is a sliding tool element, flanked by “W” and “L.”
  Click the “Auto W/L” button (Fig. 10A) to reveal a drop-down menu and select “Manual Min/Max.”
- Adjust the Min/Max slider bar maximum and minimum (left and right pegs, respectively) to fine tune the contrast on your image slices. Adjust the maximum value so that the bone or other high-density material is clearly visible and distinct but that fine structures (e.g., sutures) are distinguishable and not washed-out by too high of contrast. Adjust the minimum value so that the specimen is clearly distinct from the background (see Fig. 10B).

- Click on the drop-down menu located in the upper bar of the program window, to the right of the word “Modules:”
  - Select “Volumes” module (Fig. 10A).

#### 7. Data visualization and analyses

**Fig. 11:**
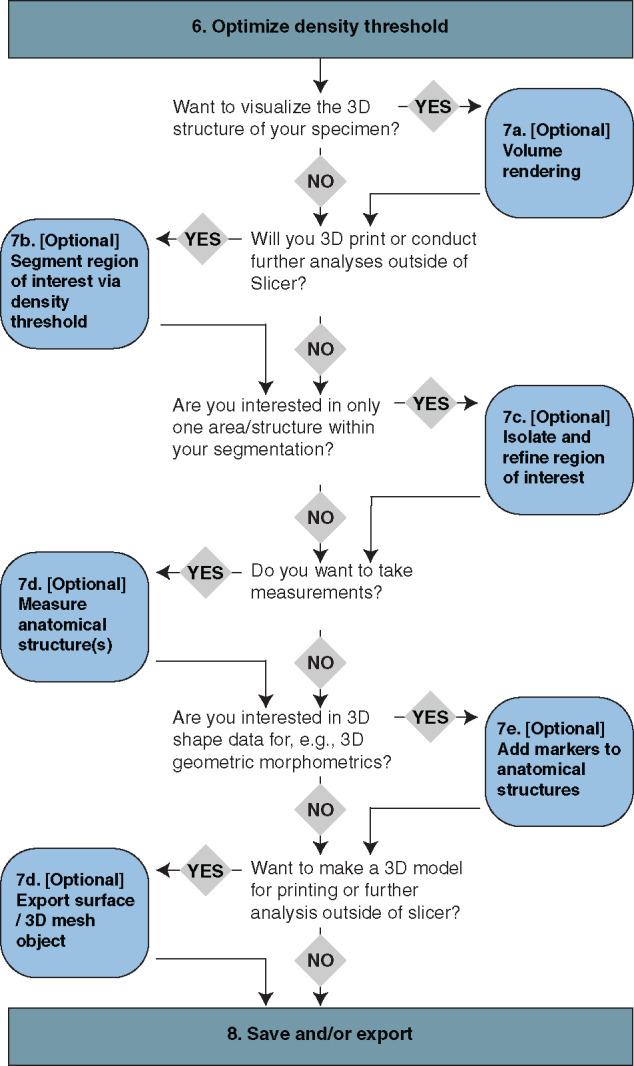
Decision tree for Workflow Steps 5–8, all performed using the program 3D Slicer. Follow the decision tree to determine which optional steps in Workflow Step 7 may be useful for your intended analyses.

There are many useful tasks and analyses available in 3D Slicer. Figure 11 illustrates a decision tree for selecting among the tasks that we have found to be most common and useful. Many of the analyses within 3D Slicer can be performed directly on the tomographic image series or on a 3D visualization of the specimen(s) therein. For users with very limited RAM and/or processing (i.e., Central Processing Unit [CPU]) speed, skipping Steps 7.a–7.c and taking measurements and landmark coordinate data directly from the slices is a way to avoid the computation and memory-taxing processes involved in 3D visualization.

**Fig. 12:**
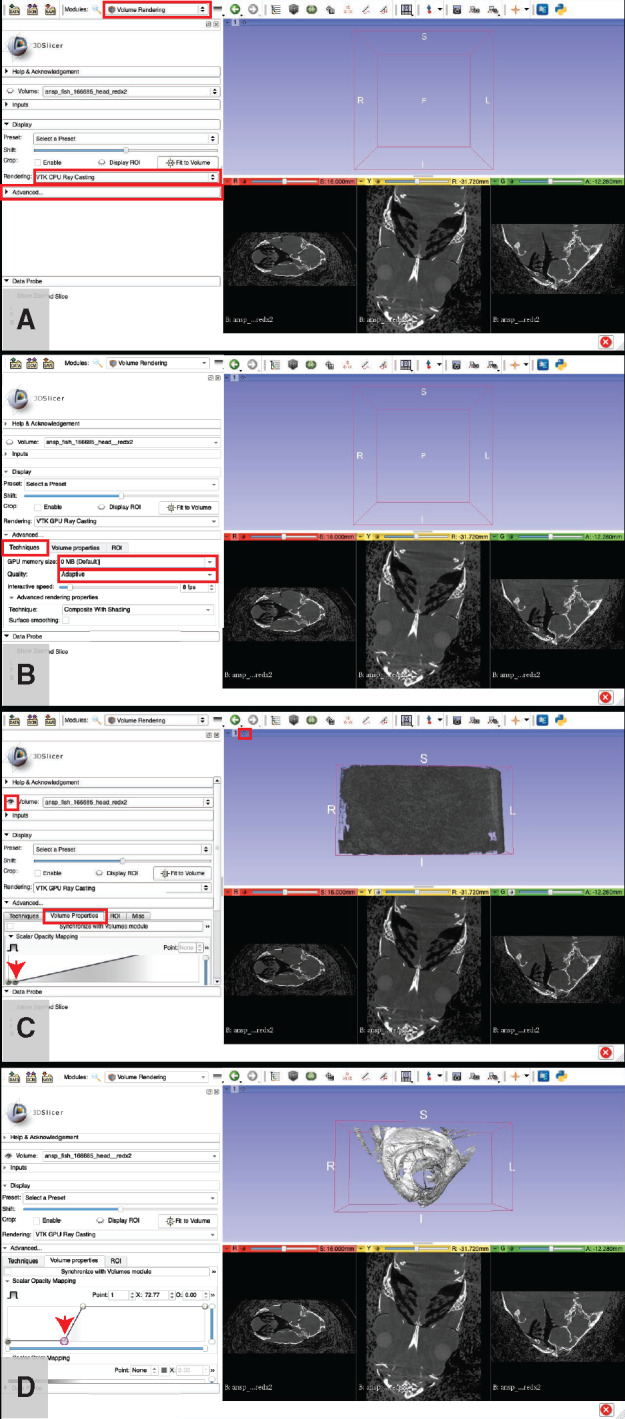
Basic volume rendering procedure. If your computer has a dedicated graphics card, change the rendering settings to use it. The “Rendering:” drop-down menu is highlighted in a red square (**A**), click it and select “VTK GPU Ray Casting.” The “Advanced…” sub-menu is highlighted in a red square (A). Click on it to expand. (**B**) The “Techniques” tab highlighted in a red square. In that tab, change the “GPU memory size:” (highlighted in red box) to match the graphics memory of your computer and change the “Quality:” (highlighted in red box) to “Normal” (B). The eyeball icon that toggles showing/hiding the volume rendering is highlighted with a red square (**C**). In the “Advanced…” submenu, click the “Volume Properties” tab (highlighted in red box) (C). Adjust the number and position of points on the “Scalar Opacity Mapping” curve so that there are four points and they create a backwards “Z” shaped curve. Adjust the position of the second point (indicated with a red arrow) until the anatomy of interest is visible, as shown in (C) (starting position) and (**D**) (final position). Adjust additional volume rendering parameters in the “Advanced” controls to fine-tune the visualization as needed. Illustrates Workflow Step 7.a i–iii.1.
      - Click on the “Quality:” drop down menu and select “Normal” (Fig. 12B).
        Note: If you wish to take a high-quality snapshot of a volume rendering (see Step 7.a.ii.3 below), you can avoid unnecessary lag time by optimizing the volume rendering parameters under “Normal” quality, then changing the quality to “Maximum” just before taking the snapshot.
      - Note: The process for checking for the presence and specifications of a dedicated graphics card varies by operating system, but this is a rigidly defined area of doubt and uncertainty and can typically be resolved with a quick Internet search.
    2. Click the eyeball icon located to the left of the “Volume” drop down menu (see Fig. 12C) to toggle whether or not the 3D rendered volume is visible. If the eye is closed, click it to open it and the volume will appear in the purple window (after some loading time).
    3. When your volume appears, it will show up as a gray block in the purple window. Click the “Center View” button in the top-left of the purple window to center the volume rendering (see Fig. 12C).
    4. For a quick visualization of the skeletons of your specimen, click the “Preset:” drop-down menu.
      - Hover your cursor over the top-left image in the drop-down menu.
        The name “CT-AAA” will appear.
      - Click this image.
      - Located immediately below the “Preset:” drop-down menu is a slider bar for adjusting the “Shift:” of the preset.
        Adjust the peg on the “Shift:” slider bar left or right until the rendering shows the skeleton of your specimen.
    5. For a customized visualization of your specimen, click the “Volume Properties” tab in the “Advanced…” sub menu (Fig. 12C).
      - Adjust the “Scalar Opacity Mapping” controls to reveal the structure(s) of interest (Fig. 12C and D).
        Tip: Add points on the opacity value curve by clicking on it. Start with four points. Select a point using the “Point:” box. Adjust the left-right position (corresponds to the density/gray scale values of your original CT dataset) of that point using the “X:” box. Adjust the opacity value of that point using the “O:” box.
        Tip: Start with four points in the Scalar Opacity Mapping graph: two on the left at the bottom of the graph (O: 0.00) and two on the right at the top of the graph (O: 1.00). Adjust the *X* position of each point as follows: Point 0, *X* = 0; Point 1, *X* = 50; Point 2: *X* = 200; Point 3, *X* = 255. Now, adjust the *X* position of Point 1 until the structures of interest are revealed (Fig. 12C and D).
      - Use the “Scalar Color Mapping” to assign colors to ranges of the opacity curve (Fig. 13A).

- **Volume rendering**: This step creates a 3D visualization of the dataset and allows the operator to assign different values of opacity and color to materials of different density. It is useful for data exploration, measuring, and counting anatomical structures (see Step 7.d below), placing anatomical landmarks (see Step 7.e below), and creating images of the anatomy (see Step 7.a.ii.5 below; see also examples in Conway et al. 2017; Conway et al. 2018). Volume renderings have been used to provide visual evidence of damage or healing to parts of the skeleton (Kolmann et al. 2020), visualize otoliths (Paig-Tran et al. 2016), assess stomach contents (Kolmann et al. 2018), and track changes in the orientation of anatomical structures across specimens (Kolmann et al. 2016, 2019; see also Workflow Step 9.d below). Volume renderings cannot be used for 3D printing other downstream processes that take place outside of 3D Slicer such as FEA. Volume rendering can be computationally taxing, especially on older machines, and some users may experience frustrating lag-times when attempting to visualize even modest-sized datasets. If your machine has a dedicated graphics card, using it will drastically reduce lag and other difficulties associated with volume rendering (see Step 7.a.ii.1 below). Alternatively, many of the same operations performed on volume renderings (e.g., measuring anatomical structures) can be performed on surface renderings (Step 7.b below), which do not tax the CPU nearly as much (but typically require more RAM than volume renderings in Slicer).
  - Click on the “modules” dropdown menu and click on “Volume Rendering.”
  - In the “Volume Rendering Module,” tweak the inputs until you can see the anatomical structures of interest (see Fig. 12):
    1. If you have a dedicated graphics card, change the rendering settings so that Slicer uses the graphics card rather than your CPU to render your data. This will increase the performance of your machine drastically for all steps related to volume rendering.
      - In the “Display” sub-menu, click the dropdown menu titled “Rendering,” and change “VTK CPU Ray Casting” to “VTK GPU Ray Casting” (Fig. 12A).
      - Expand the “Advanced…” sub-menu by clicking on it (Fig. 12A).
      - Click on the “Techniques” tab (Fig. 12B).
        Click on the “GPU memory size:” drop-down menu and select a unit of memory that is close to but not greater than that of your dedicated graphics memory. For example, if you have an “Intel Iris 1536 MB” graphics card, you would select “1.5 GB” from the drop-down menu (Fig. 12B).

**Fig. 13:**
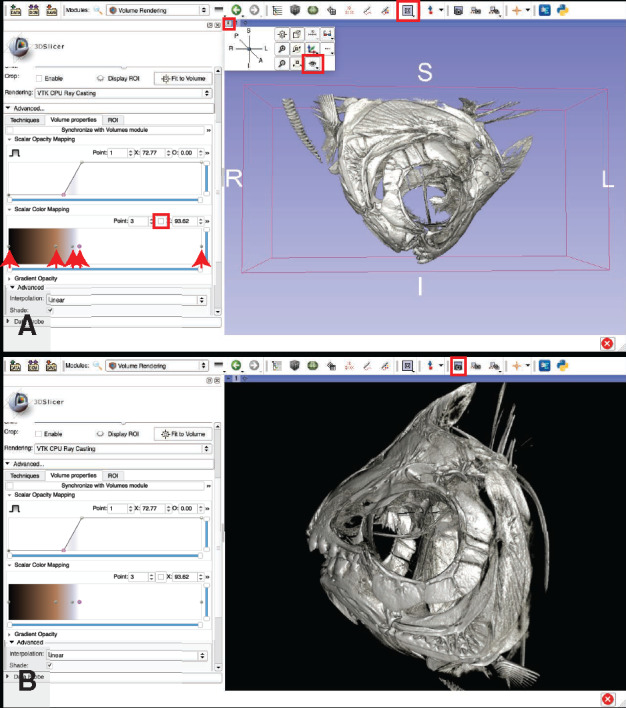
Fine-tuning a volume rendering by adjusting the color rendering of the volume (**A**) and the background view settings (**B**). Add and adjust the position of points (indicated with red arrows) in the Scalar Color Mapping graph and assign a color to each point (A). In this example, there are five points. Points 1 and 2 are assigned the color black, Point 3 is assigned the color brown, and Points 4 and 5 are assigned the color white. The view of the volume rendering in (B) has been adjusted such that the bounding box has been removed along with the axis labels, and the background color has been changed to “Black.” Illustrates Workflow Step 7.a.iii.2.
        Tip: Start with three points: one on the far left, one in the center, and one on the far right. Assign the color black to the far left (select the far-left point, which should be point “0” in the “Point:” box and click the color box immediately to the right of the “Point:” box. This will bring up the color assignment screen. Select the color black and hit the “Okay” button), gold to the center, and white to the right. Experiment with how changing the position of the center dot on the horizontal axis changes the color map on your specimen. Try adding additional points by clicking anywhere in the “Scalar Color Mapping:” graph. Experiment with different colors (and brightness values thereof) and positions for each dot until you find a scheme that you find suitable (see Fig. 13A).
    6. Tip: To further refine the 3D image, change the specimen view from “Conventional” to “3D Only” (Fig. 13A). Click the “Pin” button on the top left of the purple “3D Only” window. Click the “Eye” button to open a screen that allows you to toggle on and off the specimen bounding box and 3D axis labels (Fig. 13A). This screen also allows you to change the background color from “Light Blue” (default color), to “Black” or “White.” There are many other useful features contained in the “Pin” window. One of which is the blue and red sunglasses button, which allows the user to project the image using anagram or other specialty-glasses-enabled schemes. When you are satisfied with your view of the specimen, export an image using the camera icon (Fig. 13B). Your image will be saved by default at the resolution of your screen. To change the resolution of the image, change the “Scale factor:” to higher (increased resolution) or lower (decreased resolution) than the default value of “1.0.” When you press “OK,” your image has been taken, but not yet saved. Go to the “File” drop-down menu and select “Save.” Here you can save your snapshot by checking only the box for your labeled snapshot (see Step 8 below).
- **Segment bone or other dense material(s) of interest using a density threshol**d: This step is useful for creating 3D models of anatomy that can be used for fine-scale digital dissection (see Step 7.c), measuring (see Step 7.d), and/or placing landmarks (see Step 7.e) on anatomical structures. The segmentations produced in this step can be used to create surface renderings that can be exported as 3D meshes and used for 3D printing and/or downstream analyses in other programs (see Step 7.f). While we use a density based “threshold” to create a segmentation here, there are several other options within 3D slicer for creating segmentations. We have found the threshold-based approach to be the simplest and most accessible option, especially for new users. However, we encourage readers to explore the other options once they become comfortable with the basic steps outlined here.
  Click on the drop-down menu for “Modules.”
    1. Select “Segment Editor” module.
  Click the “Add” button to add a new segment (Fig. 14A).

**Fig. 14:**
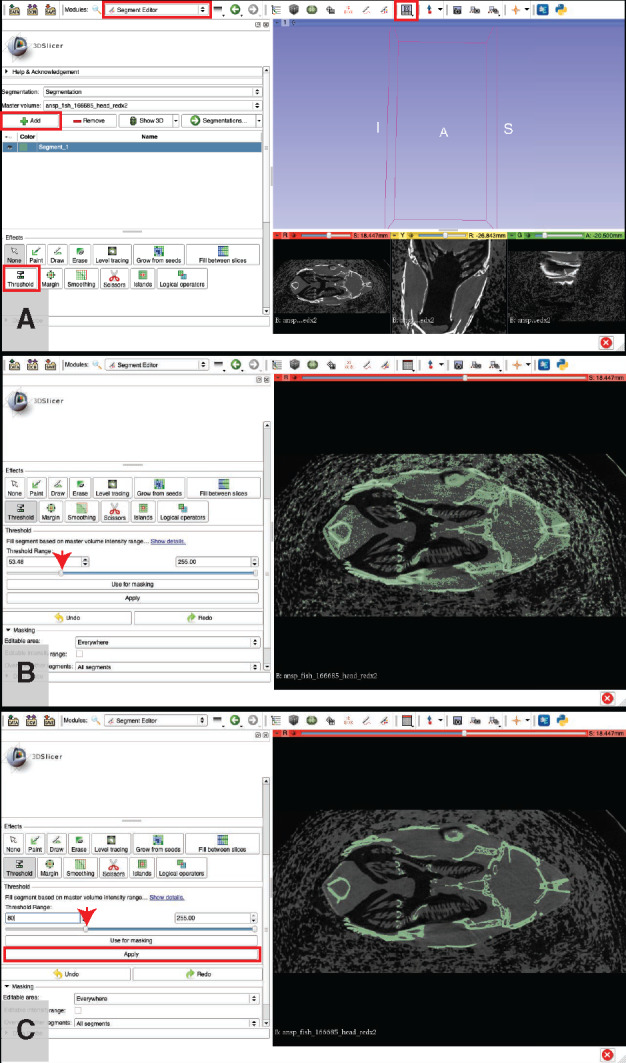
Creating a density-based segmentation of an anatomical structure of interest. Change the view (highlighted with a red box in (**A**)) to “Red slice only” after adding a new segment and clicking the “Threshold” button. Set the upper bounds of the “Threshold Range” to a value of “255.” Adjust the lower bounds (indicated with a red arrow in (A and **B**)) so that only the material of interest is highlighted. Too low of a value will capture extraneous material (B), but an appropriate value will capture only the material of interest (**C**). Illustrates Workflow Step 7.b.
    1. Keep both the default color and name of this new segment or customize by double-clicking on either one.
  Click the “Threshold” button (Fig. 14A).
  Scroll down to find the “Threshold Range:” indicator. You can adjust the lower and upper bounds of the threshold range by adjusting the left and right pegs (respectively) on the indicator bar, or by changing the values in the left and right boxes (respectively). For most applications (and/or for a starting point), set the upper bounds to the maximum value (255). Adjust the lower bounds according to the minimum density material that you wish to include in your segment. Very low values of the lower bounds will cause your segment to include lower-density material while high values of the lower bounds will result in only denser material being included.
    1. Note: While most users working with fresh or preserved specimens have little need to adjust the upper bounds of the threshold range beyond what is described above, users working with fossil data may wish to adjust the upper threshold bounds to eliminate undesirable high-density materials in the surrounding matrix. Users working with fossil data especially are encouraged to review Sutton et al. (2014).
  To get a closer look at the effect of changing your threshold range, change the view of your workspace so that you are only looking at one of the slice views of your data (Fig. 14A). The default view is called “Conventional” and includes a 3D window on top and a sagittal, coronal, and axial view below. Change the view to that of the sagittal slice (the red window below) by clicking the Slicer layout button to reveal a drop-down menu of different view options.
    1. Select “Red slice only” (Fig. 14B).
  Now we can clearly see the effects of changing our threshold range for this slice. Adjust your threshold until as much of the bone is captured (it will change color to whatever you have selected for your segment).
    1. Tip: Set your threshold value initially by lowering it until speckles of segment (as indicated by the segmentation color) begin to appear in unwanted areas of your specimen (e.g., soft tissue such as the lens of the eye if present; see Fig. 14B). Next, raise the threshold value just until all of these undesirable spots disappear (Fig. 14C). Next, check for areas in your structure of interest that are thin and adjust the threshold as necessary to ensure that all areas are encapsulated by the segment. It may not be possible to set a threshold that perfectly captures your anatomical feature of interest, but the segment can be trimmed or expanded using the eraser or paintbrush tools (respectively) to make fine adjustments to the area included in the segment and match it to your structure of interest (see Step 7.c below).
  Press the “Apply” button (Fig. 14C).
- **Isolate regions of interest from segmentation**: This step is useful for isolating small and/or complex structures such as individual bones from a skeleton, or a single specimen from scan data that contain multiple specimens.
  Visualize the 3D structure of your segment.
    1. Select “Conventional View” (Fig. 15A).

**Fig. 15:**
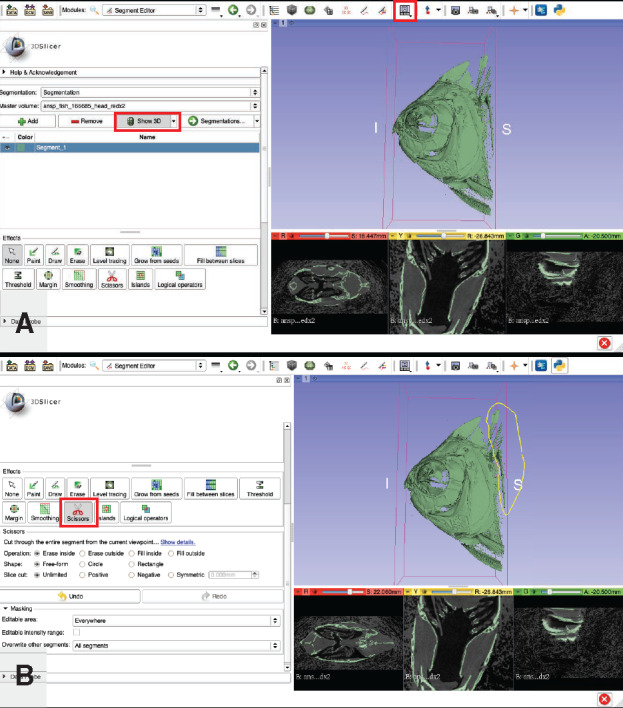
Isolating a region of interest from a segmentation. Using the 3D view of the segmentation (**A**), extraneous structures are selected and eliminated using the “Scissors” tool (**B**). Illustrates Workflow Step 7.c.
    2. In the “Segment Editor” module, click the “Show 3D” button (Fig. 15A).
    3. To reposition your specimen/segment in the 3D window, select the “None” tool in the “Effects” section. Using your left mouse button will rotate specimen, holding down the “Shift” key while dragging with the left mouse button with reposition your specimen, the right mouse button will zoom in and out.
  Select the “Scissors” button in the “Effects” section (Fig. 15B).
    1. This tool can be used on either the 3D view or any of the slice views and has several options available, perhaps the most useful for us are the “Erase inside” and “Erase outside” options under the “Operation:” section. Keep in mind that the erasure applies to the entire image stack, so use this tool carefully, especially when used within a slice view.
      - Note: To remove unwanted areas of the segmentation from only a single slice, select the “Erase” button in the “Effects” section. This tool can be time-consuming to use but is invaluable for fine-scale cleanup of a segmentation.
  To erase part of your segment that is not of interest, click the “Erase inside” option, then encircle the undesired region with the scissor tool (Fig. 15B).
    1. Note: If you have a region of interest that is fairly uniform in shape, it may be useful to start the cleanup process by first using the scissor tool with the “Erase outside” option selected before switching to the “Erase inside” option for further cleanup.
- **Measure anatomical structures**: This step can be performed in the 3D view (on either a volume or surface rendering) or in any of the slice views.
  Go to the “Annotations” module from the module drop-down menu (Fig. 16A).

**Fig. 16:**
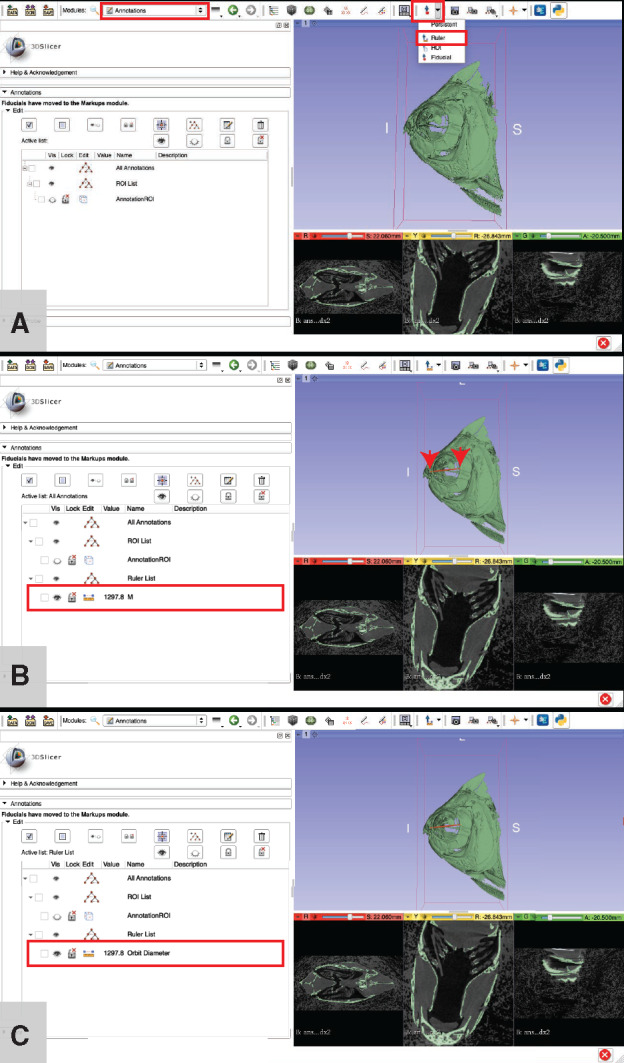
Measuring anatomical structures using the “ruler” tool in 3D Slicer. Use the ruler tool (**A**) to place two points on a structure of interest (each point indicated with a red arrow in (**B**)). To increase the visibility of the line drawn by the ruler, click the ruler icon in the “Edit” column, then expand the “Advanced” submenu by clicking on it, next click the “Lines” tab and adjust the “Width” value using either the slider bar or by inputting the desired number directly. To keep organized, we recommend giving each measurement a descriptive name (**C**). Illustrates Workflow Step 7.d.
  Select the “Ruler” tool from the cursor tools drop-down menu (Fig. 16A).
  Make sure that the entire structure is visible before attempting to measure it.
    1. Tip: If it is not possible to view the entire structure when placing the measurement points, place the points as close as possible to where they should be, then change views and move the points into their correct position(s) by clicking and dragging with the cursor.
  Click on each end of the structure that you intend to measure (Fig. 16B).
    1. The value of the measurement (i.e., the length) will appear next to a line connecting the two points of your measurement. The measured length will also appear in the “Annotations” box, next to the measurement (it will be given a default name) in the “Value” column (Fig. 16B).
    2. Note: the measurement will be indicated in the units that you specified in Step 3.a. If you followed our example, this unit is “pym” (see Step 5.f if 3D Slicer reports this value in an undesirable unit). If you are interested in comparing this measure to measures taken other units (e.g., mm), you will need to convert your measures to a common unit. For example, we measured the orbit diameter of the pacu specimen as 1297.8 pym, which is equivalent to 12.978 mm. The orbit of the sculpin specimen is ∼308.2 pym, which is equivalent to 3.082 mm.
  Optional: If you will be making multiple measurements, you can keep track of them in the “Annotations” window. You can change the name of a measurement to reflect what it is measuring (e.g., “Orbit Diameter”), hide measurements from the 3D viewer, delete measurements that were not satisfactory, etc. (Fig 16C).
- **Adding markers to anatomical landmarks of interest**: This step can be used for capturing and exporting 3D coordinate values for anatomical landmarks. These values can be used to calculate distances between the landmark points and/or used in geometric morphometrics studies. Landmarks can be placed in either the 3D view (on either a volume or surface rendering) or in any of the slice views. Users interested in advanced landmark placement and analysis (including the use of sliding semi-landmark curves, etc.) are encouraged to explore the “SlicerMorph” extension for 3D Slicer (Rolfe et al. 2020; slicermorph.github.io).
  Click on the “Modules” dropdown menu and click on the “Markups” module (Fig. 17A).

**Fig. 17:**
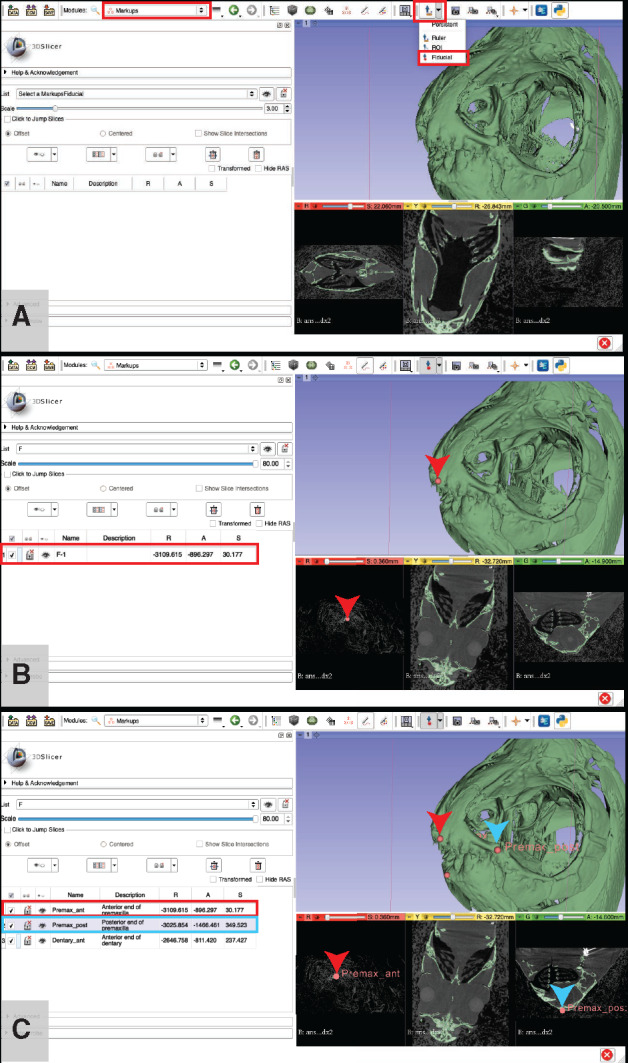
Placing markers on anatomical landmarks using fiducial points in 3D Slicer. Use the “Markups” module to organize and annotate the “Fiducial” points (**A**). Users can adjust the size of the fiducial points to a size that best suits their needs by using the “Scale” indicator, either sliding the peg left or right on the “Scale” bar or by entering a number directly into the box on the right side of the bar. For the purposes of our demonstration, we use a scale of “80” for maximum visibility. After a point is placed (indicated with a red arrow in (**B)** and (**C**)), replace the default name (information for that point highlighted with a red box in (A) with a descriptive one (highlighted with a red box in (B) and do this for each new point (two additional points are shown in (C): one indicated with a blue arrowhead and the corresponding information highlighted with a blue box, the other with no arrowhead or box) to maintain organization. Points can be placed on either the 3D view of the segmentation or in any of the slice views. The points “Premax_ant” and “Premax_post” are visible in both the 3D and slice views and indicated with a red and blue arrowhead (respectively) in both. Illustrates Workflow Step 7.e.
  Click the Marker dropdown menu to select the “Fiducial” option (Fig. 17A).
    1. Tip: Use the 3D and 2D views to ensure your markers are in the right place.
    2. Tip: To center all views around a particular marker, right-click that marker in the table to the left and select “Jump Slices” to go to the corresponding slices in the RYG view and “Refocus all Cameras” to center the 3D view around that mark.
  Click on your point of interest in either the 3D or any slice view to place a landmark (Fig. 17B).
    1. A description of the landmark will appear in the “Markups” module window, which includes an auto-generated name for the landmark as well as its coordinates.
      - Note: Slicer was created to work with medical data. The coordinate system, “RAS,” is short for the human anatomical coordinate system (R: left toward Right, A: posterior toward Anterior, S: inferior toward Superior). More info here: https://www.slicer.org/wiki/Coordinate_systems.
    2. Tip: The “Scale” slider bar controls the size of the markers. Slide it to a size that is easy for you to see on whichever window you are using to mark anatomy.
  Tip: Give each landmark a descriptive name by double-clicking on each auto-generated name and replacing it (Fig. 17C).
- **Export surface/3D mesh object**: This step exports the segmentation that was created in Workflow Step 7.b as a 3D mesh object that can be saved and read into other software packages. Three-dimensional mesh objects are the basis for many downstream applications. They can be 3D printed (or milled) from ceramics, plastics, or even metal and used to test how shape affects performance of certain morphologies like teeth, jaws, or filtering apparatuses (Kolmann et al. 2015; Cohen and Hernandez 2018; Divi et al. 2018). Mesh objects can also be used for gathering 3D geometric morphometrics data (e.g., Sherratt et al. 2014, 2019; Buser et al. 2018; Evans et al. 2019a, 2019b; Selig et al. 2019).
  Go to the “Segmentations” module (Fig. 18A).

**Fig. 18:**
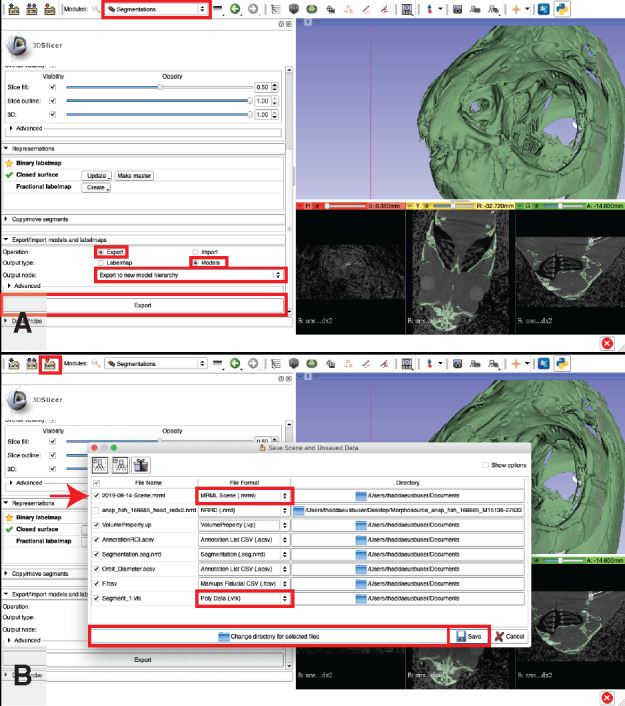
Exporting the 3D segmentation as a surface mesh (**A**) and saving data files (**B**). We recommend changing the default surface mesh format (highlighted with a red box in (B)) from the default.VTK to a more standard format, such as .OBJ, before saving. The “Scene” file line is indicated with a red arrow and the file format drop down menu for both the “Scene” and the 3D surface mesh are each indicated with a red box (B). Illustrates Workflow Steps 7.f and 8.
  Scroll down on the left panel and click the “Export/import models and labelmaps” drop down menu (Fig. 18A).
  For “Operation:” select, “Export” (Fig. 18A).
  For “Output type,” there are two options:
    1. “Labelmap” exports the segmented area as a labeled area in the 3D space of your image stack. This "map" can then be used to isolate areas of your image stack (e.g., using the "Mask Scalar Volume" module).
      - This format lends itself to plugins such as BoneJ for ImageJ (Doube et al. 2010; http://bonej.org/) to calculate shape properties such as second moment of area of the 3D shape.
    2. “Models” exports a 3D surface model, which is composed of points (vertices) in 3D coordinate space that are connected to one another by lines (edges). The triangles formed between the lines connecting each point are called “faces” and can be assigned properties such as color (the default color in 3D Slicer is “white”). This function is what you will use to generate a surface model for eventual 3D printing.
  Change the “Output type” to whichever suits your needs or whichever you wish to export first (Fig. 18A).
  Click the “Export” button (Fig. 18A).

#### 8. Saving data

Click the “Save” button in the upper left corner of the 3D Slicer window (Fig. 18B).
  Click the “Change the directory for the selected files” button at the bottom of the window (Fig. 18B) and specify a directory for storing your files.
  If you added landmarks, custom volume properties, ROI crops, segments, or settings and would like to change the name(s), you can do so here.
  If you exported a 3D surface model, the default file type is “.vtk.” We recommend changing the file type to a more standard format, such as “.obj,” “.stl,” or “.ply” (Fig. 18B). These file types are the standard formats for 3D printing or refining models prior to 3D printing (using programs like MeshLab).
  Check all boxes that contain files and settings you wish to save.
  Tip: If you would like to save all of your files as a single file that you can easily share with colleagues, find the “Scene.mrml” file (Fig. 18B).
    1. Click on the “File Format” drop down menu for the scene file and select “Medical Record Bundle” to save all of your files and settings under one scene file. This format facilitates easy sharing of data, but is not recommended for archiving your work.
      Note: It is also possible to create a medical record bundle by clicking the “Create a Medical Record Bundle containing this scene” button, which is shaped like a wrapped present and is located in the upper left corner of the “Save Scene and Unsaved Data” pop up window. Clicking this button will automatically change the scene file type to “Medical Record Bundle” or “Medical Reality Bundle” depending on your Slicer version and dependencies.
    2. Note: This operation will not save any segmentations as separate 3D surface models, or export any other file separately (e.g., measurements, landmark coordinates, etc.) so if you intend to do so, perform Step 8.i.4 (above) and 8.ii (below) without creating a Medical Record Bundle. You can perform both of these tasks, but they will need to be performed separately.
Press the “Save” button (Fig. 18B).
  Note: If you created a “Medical Record Bundle,” this step creates a single document containing all work that can be shared with collaborators and/or reopened by dragging and dropping the file into a new 3D Slicer window. If you did not create a Medical Record Bundle, this step saves each file separately.

#### 9. Example analyses

- **Measuring traits associated with a functional morphology** (Supplementary Video S1): This video shows the complete workflow necessary to measure the anatomical traits examined in Buser et al. (2019), using the same CT data analyzed for one specimen included in their study. This includes downloading a CT image stack for a sculpin specimen (*Cottus asper)* from morphosource.org (MorphoSource ID M-15632), preparing the data in Fiji, visualizing and segmenting the skull, placing anatomical landmarks, measuring, and exporting and saving data. Workflow Steps demonstrated: 1, 2.a–c, 3.a–b, 3.d, 4.a–b, 5.a–e, 6, 7.a–e, and 8.
- **Digitally isolate the oral jaws of a fish** (Supplementary Video S2): This video shows how to digitally isolate an anatomical structure of interest from the scan of a larger object. The example uses a pinfish (*Lagodon rhomboides*) specimen downloaded from MorphoSource (MorphoSource ID: M16875-31342), but the method could be easily extrapolated to any other organism of interest. Steps include visualizing, cropping, rotating, segmenting, and digitally dissecting a segmentation using a CT image stack. Workflow Steps demonstrated: 7.a, 7.b, 7.c, 7.f, and 8.
- **Isolate a region of interest using local thresholding and semi-automated segmentation** (Supplementary Data S1): This supplementary workflow starts with product of Workflow Steps 1–3 (a reconstructed scan, either edited or not), and shows an alternative approach to Steps 7.b and 7.c using a CT image stack of a specimen of *Oodinus* sp. (Carabidae; MorphoSource ID M47304-85911). This approach is potentially useful for researchers performing a high number of segmentations (either of the same structure on multiple specimens, or, especially, several structures within a single specimen), as several steps are semi-automated and thus reduce operator time per segmentation. Additional software required: Segmentation Editor and 3D Viewer (pre-installed in Fiji distribution of ImageJ), Biomedisa (Lösel and Heuveline 2016).
- **Simple Tricks and Nonsense: collect data from simple visual tools** (Supplementary Data S2): This supplementary workflow shows how CT data can be used to quickly and easily visualize anatomical structures for rapid assessment. Workflow Steps demonstrated: 7.a.
- **Reduce file size of a 3D model and convert file type** (Supplementary Video S3): This video uses the 3D segment model that was exported and saved in Workflow Steps 7.f and 8 (respectively) and uses the program MeshLab to reduce the complexity and file size of the 3D model and convert the file type from OBJ to PLY. The video then demonstrates reading the PLY file into the R statistical environment. The file reduction and reformatting tasks are often necessary for preparing 3D models for 3D printing or for use in programs for downstream analyses, and there are a variety of morphometric analyses that can be performed on 3D data in R (e.g., collecting 3D landmark data). MeshLab can also be useful in reflecting features if specimens are asymmetrical/damaged. Additional software required: MeshLab, R (R Core Team 2019), RStudio (R Studio Team 2018), and the R package, “geomorph” (Adams and Otárola-Castillo 2013; Adams et al. 2019).

## Conclusion

Here, we have outlined steps that will help a researcher begin their journey through the CT galaxy. This workflow was developed through our own exploration and should provide researchers a means to test many of their own hypotheses without getting lost in the space of possibilities. Beyond its use in the fields of comparative anatomy, evolution, and functional morphology that we have highlighted, this workflow could be easily adapted to fields such as paleontology, paleoanthropology, archaeology, museums and heritage, biomedical research, mineralogy, and geology, to name but a few. As with all great frontiers, there is much more to explore, seek out, and more places to boldly go in years to come.

### A CT on the edge of forever

The potentially unlimited lifespan of CT data makes them useful not only to the researcher(s) who made the initial scan, but also to future researchers who may ask questions that the original scanner would never have considered. Collections and researchers should consider these future applications when choosing the license under which they share data, as retaining a strict copyright on the scanned image and its derivates (as is the standard policy of some prominent museums) may severely limit the ability of future researchers to reuse scans or data resulting from those scans. While it is beyond the scope of this article to discuss the nuances of the many types of licenses available, we note that many data aggregators (including GBIF and VertNet) recommend some version of a Creative Commons license for biodiversity data, and suggest that other options stifle reuse and may even be legally unenforceable. When the data are made open access under a Creative Commons license, the number of future researchers that may examine them, and thereby the number of future studies that may come from them are virtually limitless. Until such licenses become ubiquitous, researchers seeking to reuse scans from data aggregators should check carefully the terms under which each digitization has been shared, and take pains to request permission for reuse for any scans published under a restricted license.

There are many potential applications of CT data that are only beginning to be explored by natural historians. For example, the ImageJ extension BoneJ can be used to calculate biomechanical attributes (e.g., second moment of area) of anatomical structures (see Workflow Step 9.f above; Rutledge et al. 2019). The use of CT-based models to gather 3D geometric morphometrics data has been widely embraced (see Workflow Steps 9.e and 9.f; Zelditch et al. 2012; Sherratt 2014), and offers important advantages over more traditional 2D geometric morphometrics (Buser et al. 2018). Such models can also be used to construct digital models for biomechanical analysis through applications such as FEA (Jaecques et al. 2004; Oftadeh et al. 2016; Stayton 2018). Pairing CT with other bio-imaging techniques like histology or material testing has great potential in the visualization and interpretation of complex anatomies, as well as making sure digital models (i.e., FEA) are accurately mimicking structural complexity (Jayasankar et al. 2017; Lessner et al. 2019; Seidel et al. 2019; Wilken et al. 2019). Another application of CT data that has great potential for research in comparative biology is the ability to estimate bone mineral density (Cann 1988; Schreiber et al. 2014). By including samples of materials with known density in their scans, researchers can estimate the density of the bone mineral in their specimens and compare the relative density of anatomical structures across large numbers of specimens. The use of contrast-enhancing elements for staining soft tissue, particularly when paired with histology, is also on the forefront of CT-based natural history and functional morphology studies (see Workflow Step 9.c; Pauwels et al. 2013; Descamps et al., 2014; Gignac et al. 2016; Hongjamrassilp et al. 2018). Finally, there is more to explore even in the way that researchers gather CT data. A recently described method of ultra-high resolution CT-based 3D reconstruction (“X-ray histotomography,” see Ding et al. 2019) shows great potential for expanding the field even further.

Researchers, educators, and enthusiasts can use the tools, techniques, and demonstrations provided in this workflow to acquire, process, and analyze the great wealth of CT data that is being shared over the Internet. While we concede that we are not the guardians of the one true way of navigating the CT galaxy, we do think that our workflow will save users a lot of time, and hopefully keep them from giving up and going mad. Above all, we hope that our approach will reduce panic, and help readers launch their own galaxy quests. Anything less would be illogical.

## Supplementary Material

obaa009_Supplementary_Data
