# Supplementary material for: The Natural Historian's Guide to the CT Galaxy: Step-by-Step Instructions for Preparing and Analyzing Computed Tomographic (CT) Data Using Cross-Platform, Open Access Software
Source: Integr Org Biol. 2020 Apr 10;2(1):obaa009. doi: 10.1093/iob/obaa009 (PMC7671151; doi:10.1093/iob/obaa009)
Supplement: obaa009_Supplementary_Data [file obaa009_supplementary_data.zip › Supplementary Data 1 Step 9C_revision_200307.docx]

**SUPPLEMENTARY DATA 1 STEP 9c: Isolate region of interest using local thresholding and semi-automated segmentation:**

This approach is of potential use if there is little contrast between your structure of interest and other parts of your specimen, and/or if your structure of interest is a complex shape that is especially difficult or time consuming to cut away from the rest of your specimen using manual tools in 3D Slicer. Try this procedure if the results of threshold-based or manual segmentation in 3D Slicer are incomplete, messy, or of inadequate detail. In the example shown in the screenshots, the pixels corresponding to the exoskeleton of the beetle specimen are similar in intensity to the internal muscle and gut tissues. This lack of contrast makes it impossible to isolate (based only on a global threshold value) the exoskeleton exclusive of soft tissue for downstream analyses.

Compared with generating a surface rendering based on global thresholding and then cleaning it up manually, the method of segment isolation presented below is less dependent on human decision making, and potentially easier to replicate. Results of the segmentation algorithm (step ii) depend only on the original grayscale image stack and the labels file supplied; the variables that are up to the user are which slices to label and how many.

In most cases, the following steps will allow you to generate a surface model containing labeled (differently colored) segments you want to use for downstream analyses, and nothing you don’t want included.

1. **Isolate region of interest using local thresholding and semi-automated segmentation:**
   - 1. Local thresholding and segment labeling in ImageJ:

**Objective:** Build a “labels” file that includes labeled (colored) selections of a few slices of the region(s) of interest that you want to isolate from the rest of your scan. This file will show the segmentation algorithm (step ii) which parts of your scan to pay attention to, and which parts can be ignored. Rather than selecting pixels from all slices at once based on one threshold level, you will select pixels from within a limited area, one slice at a time (and only for a few slices).

- - - 1. Make sure the [Segmentation editor](http://132.187.25.13/home/?category=Download&page=SegmentationEditor) plugin for ImageJ is installed. This should be built in if you are using the Fiji distribution of ImageJ (Schindelin et al 2012).
      2. Open the original image stack in ImageJ, then open the “Segmentation Editor: Plugins,” select “Segmentation Editor,” and click “Segmentation.” A new version of your image stack will appear in the editor window, and a second window will show a blank (for now) stack of the same size (same number of pixels and slices) to which labels will be added.
      3. Convert the image stack to 8-bit by clicking “Image,” clicking “Type,” and selecting “8-bit.”
      4. Create a label with a unique color for each structure you want to segment: To add a new label (for example, one for head, one for prothorax, etc...), right click on one of the labels in the Segmentation Editor window (Figure 1A) and select “Add Label”, then rename it and change its color. By default, you’ll start out with two labels: one background/“exterior” (black) label and a foreground/“interior” (red) label; leave the exterior label black. You can rename and recolor the interior label, then create any other labels you want.
      5. Choose a slice that captures a segment you would like to label, then roughly outline the object (Figure 1B) using the brush selection tool (Figure 1C, found under the oval/elliptical area selection tool). Better to err on the side of including too much than missing pixels you want to include. Don’t waste time refining (removing pixels from) the selection at this point.
      6. Refine the selection by local pixel value using the Threshold tool (the “T” button under Tools in the Segmentation Editor window). Try a minimum value of around 200 and maximum of 255 to select the brightest pixels within your selection. Click the box beside “show binary” to preview the results (Figure 2A). Click Okay when the threshold captures the pixels of interest (Figure 2B).
         1. You can further refine the selection and get rid of unwanted inclusions by holding down the alt key while using the selection brush, or start holding down the brush while outside the selection. To change the brush diameter, double click the selection brush button. This step really matters because these selections will serve as the seed images for interpolation later on. It’s convenient, but not necessary, to use a pen tablet for this. If your selection disappears, go to “Edit,” click “Selection,” and select “Restore Selection” to continue editing.
      7. Once your selection is defined, click the label you want to assign to that selection, then click the “+” button under Selection in the Segmentation Editor window (Figure 3A) to add the selection for the current slice to the labels file (Figure 3B).
         1. Note: sometimes, ImageJ forgets your refined selection and labels your rough outline when the “+” button is clicked! If this happens, go to “Edit,” click “Selection,” and select “Restore Selection” and then go to “Edit,” click “Selection,” click “Make Inverse” and then add the label. This can cause problems when you’ve already labeled two or more parts of the slice. Saving frequently and starting each slice with the easiest structure to label will reduce the amount of work lost in case this issue occurs. Scrolling up and down a few slices before returning to the working slice and adding a label seems to reliably prevent ImageJ from “forgetting” the selection.
      8. Continue adding labels to the slice until everything you want visible in the final reconstruction is included. To remove part of a label for a slice (for example, a stray “island” that was accidentally included), just paint those pixels with the selection brush and assign them to the background (with background/black selected in the Labels panel, press the “+” button).
         1. Note: The more slices you label, the more information you’re providing to the segmentation algorithm. The minimum number of slices you should label depends on the size and complexity of your object—It may take some trial and error to figure this out.
      9. Before exporting, note that *within any slice you choose to label, everything you want to be included in the segmentation must be labeled*. Biomedisa will interpret any unlabeled pixels in a labeled slice as background (even if those pixels are a continuation of a structure that is labeled in another slice).
      10. To save progress so that the labels file can be added to later (highly recommended), save the labels file: go to “File,” click “Save As,” and select “AmiraMesh.” To reload: open the full 8 bit image stack, then the labels file: select “File,” click “Import,” click “Amira” and select the Amira file. Then, select the window with the full image stack and open the segmentation editor plugin.
          This solution was found in a 2017 post by Kristen Scaplen on the Scientific Community Image Forum at forum.image.sc: <https://forum.image.sc/t/save-progress-in-segmentation-editor/7039/6>
      11. Export the labels file as an 8-bit tiff, and also save an 8-bit version of the original image stack.
    1. Semi-automated segmentation in Biomedisa:

Objective: Generate a file with full reconstructions of the segments you want, and nothing you don’t want. Biomedisa (Lösel and Heuveline 2016) is automated in that it uses a random walk algorithm to reconstruct segments, but is also user-led in that it only fills in the gaps between/around the labeled slices that you provide it. It’s a browser-based app, so there is nothing to download. Two files are needed: your image stack and a label file. They must be 8-bit and their dimensions must match exactly (same number of pixels and slices).

- - - 1. Register at <https://biomedisa.de/register/>
      2. Navigate to the “App” tab and upload the image stack and label file. Make sure beforehand that both the image stack and label file are 8 bit (or use Biomedisa’s “Convert to 8-bit” tool when uploading).
      3. Click “Start”.
      4. When the segmentation process finishes (likely in less than 10 minutes), download the results using any of the post-processing options (e.g. regular, cleaned, fill holes, smooth). You can preview how the algorithm interpolated your labels through all slices using the “2D” option, which will open the stack in your browser.
      5. If you open the final project tiff stack in ImageJ, it will all look black because the value of your label is set to 1. Check the image stack histogram by going to “Analyze” and selecting “Histogram” and look at the maximum value. Adjust the contrast going to “Image,” clicking “Adjust,” clicking “Brightness/Contrast” and selecting “Auto” or bring the maximum value down to whatever that is in the histogram.
      6. Open the results as an image stack or with the 3D viewer (Schmid et al, 2010) in ImageJ: go to “Plugins” and select “3D Viewer.” On the “Display as” dropdown menu, select Surface. Select a color and a threshold value in between the values of the background and your darkest label. Use a resampling factor of 2 or more to speed up visualization (at the expense of resolution).
    1. To quickly record a 360˚ rotation video of your specimen using the 3D Viewer in ImageJ (other options for taking a snapshot or recording an animation are in the “View” menu):
       1. Position your specimen by clicking and dragging in the 3D viewer window
          (there is often a considerable lag with larger files—in this case, clicking and dragging without releasing the click seems to work better than making short adjustments).
       2. Add or remove coordinates and the bounding box under Edit.
       3. Go to “View” and select “Change Animation Options” to adjust plane of rotation and degree interval.
       4. Go to “View” and select “Record 360 degree rotation”
          A new window titled “Movie” will open.
       5. With the “Movie” window selected, go to “Image,” click “Stacks,” click “Animation,” and use “Animation Options” to adjust frame rate and which frames to include.
       6. Optionally convert from RGB by going to “Image,” clicking “Type,” and selecting “8 bit,” then “Save As” and selecting “Animated GIF.” It may take several minutes to write to the file. Open it in ImageJ or a browser window to check that the export was successful.

**REFERENCES:**

Lösel, P. and Heuveline, V. (2016) [Enhancing a diffusion algorithm for 4D image segmentation using local information](http://proceedings.spiedigitallibrary.org/proceeding.aspx?articleid=2506235). Proc. SPIE 9784, Medical Imaging 2016: Image Processing, 97842L, doi: 10.1117/12.2216202.

Schindelin, J.; Arganda-Carreras, I. & Frise, E. et al. (2012) "Fiji: an open-source platform for biological-image analysis", Nature methods 9(7): 676-682, PMID 22743772, doi:10.1038/nmeth.2019

Schmid, B., Schindelin, J., Cardona, A., Longair, M. and Heisenberg, M., 2010. A high-level 3D visualization API for Java and ImageJ. BMC bioinformatics, 11(1), p.274.

**FIGURES:**


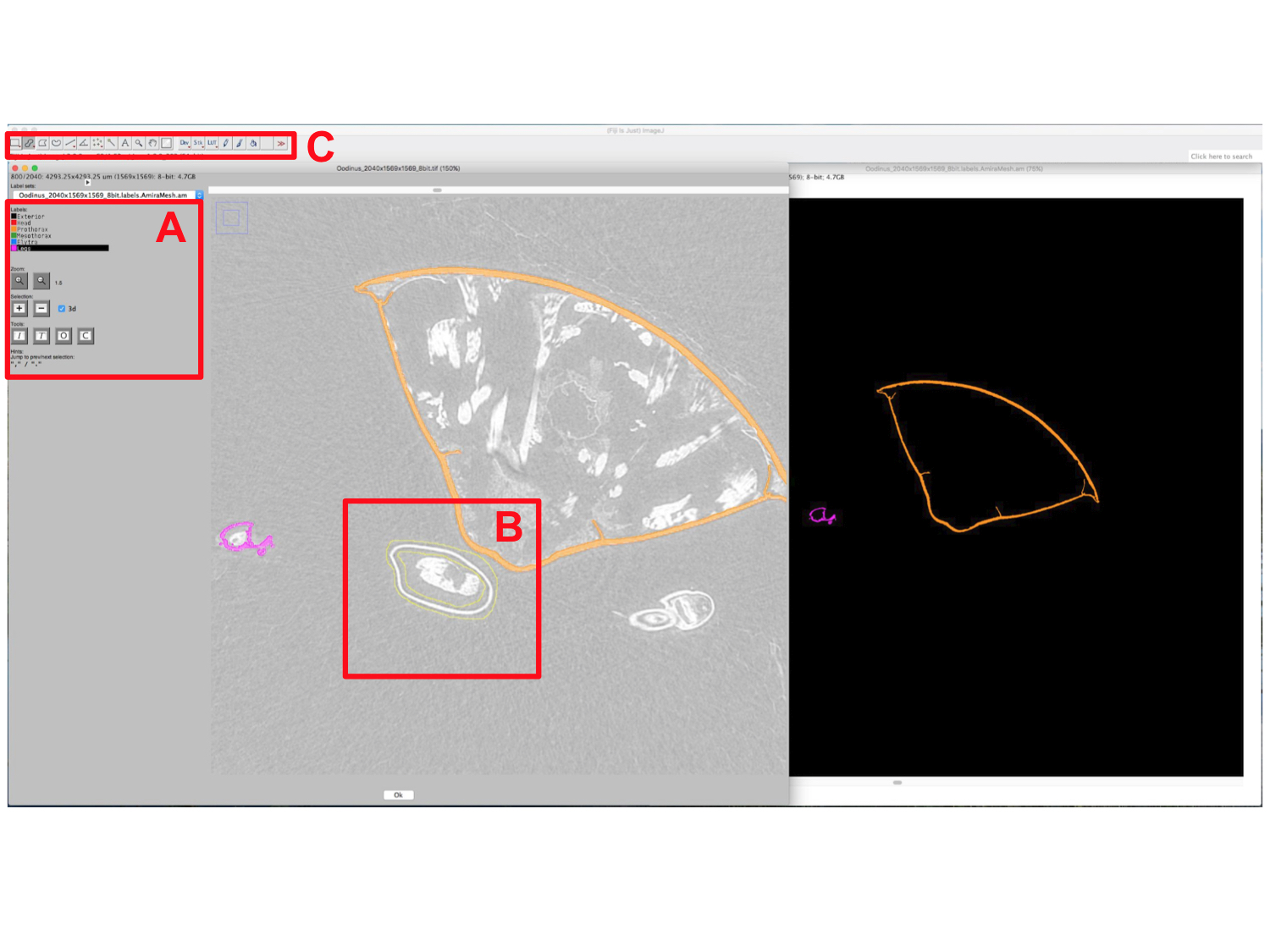


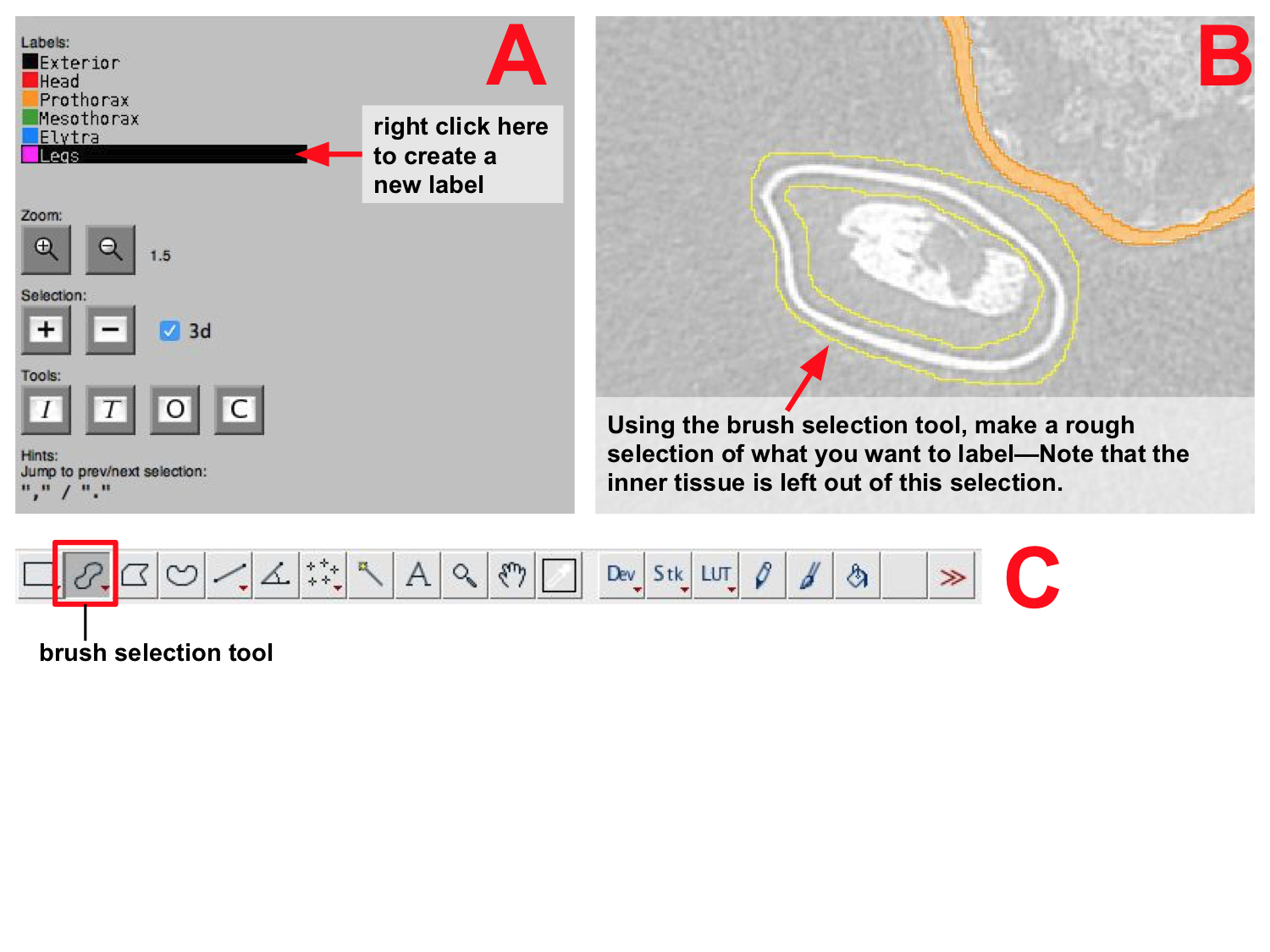


**Figure S1-1:** Basic tools in ImageJ segmentation editor.


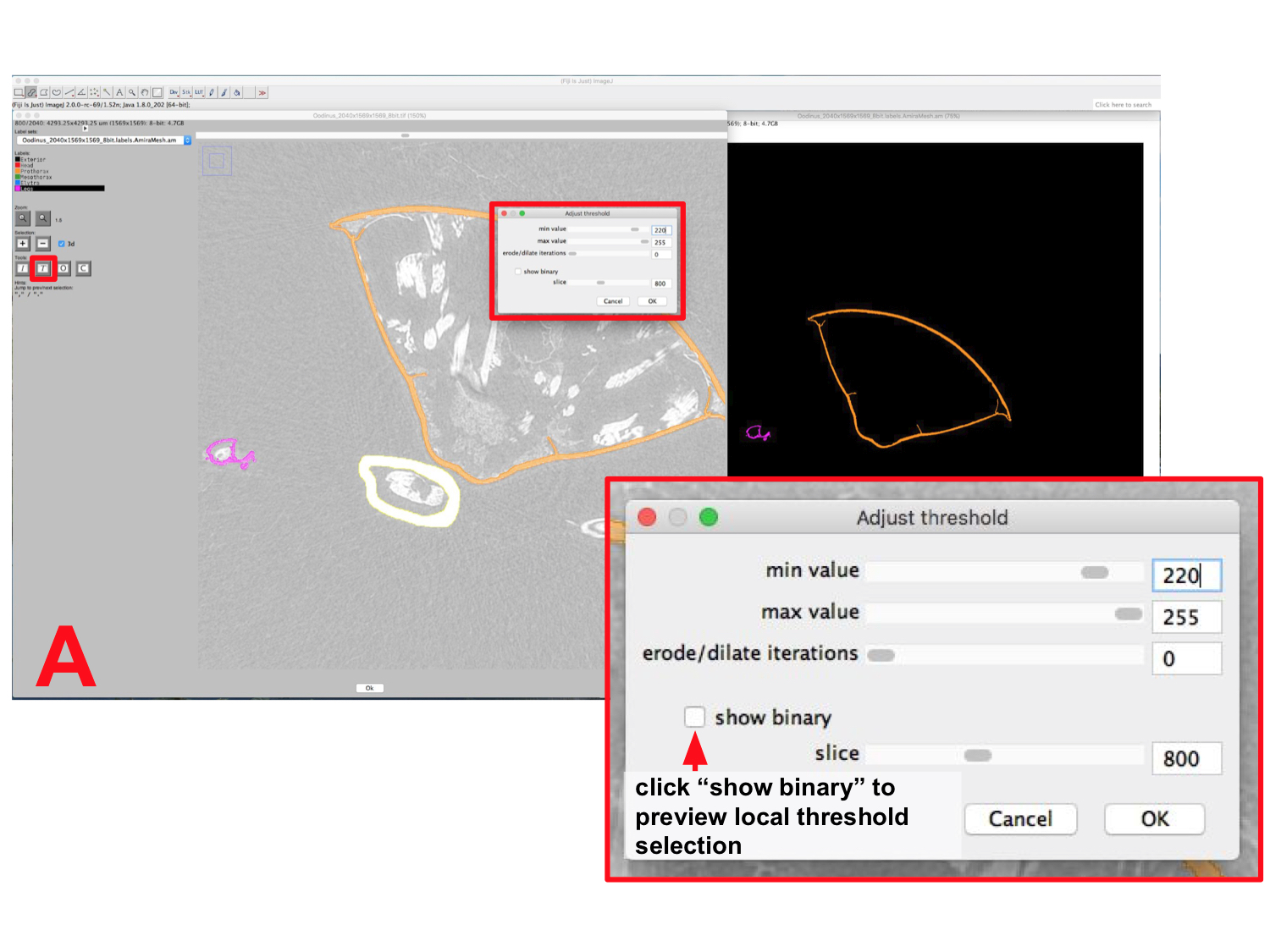


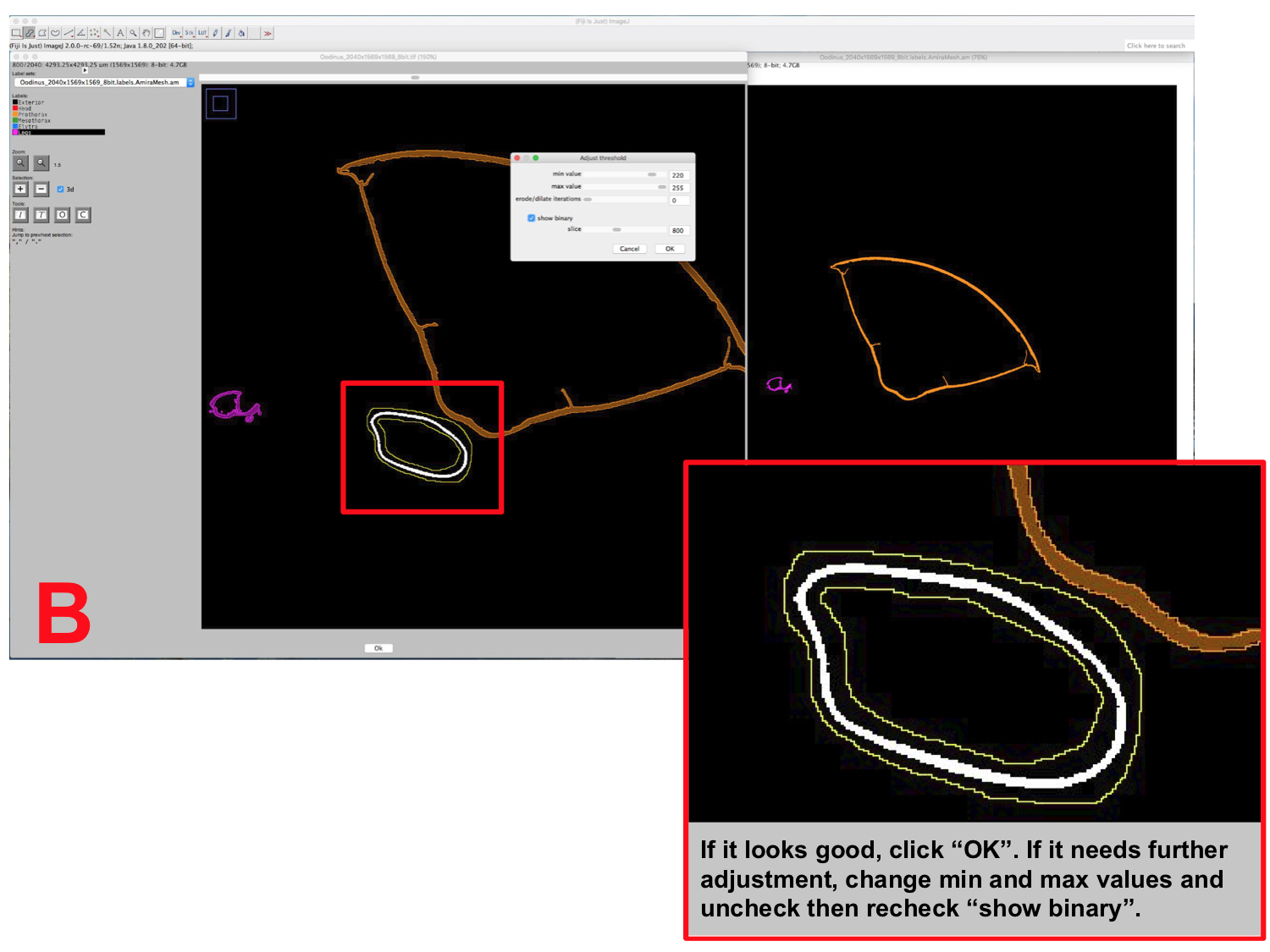


**Figure S1-2:** Adjusting threshold within your rough selection.


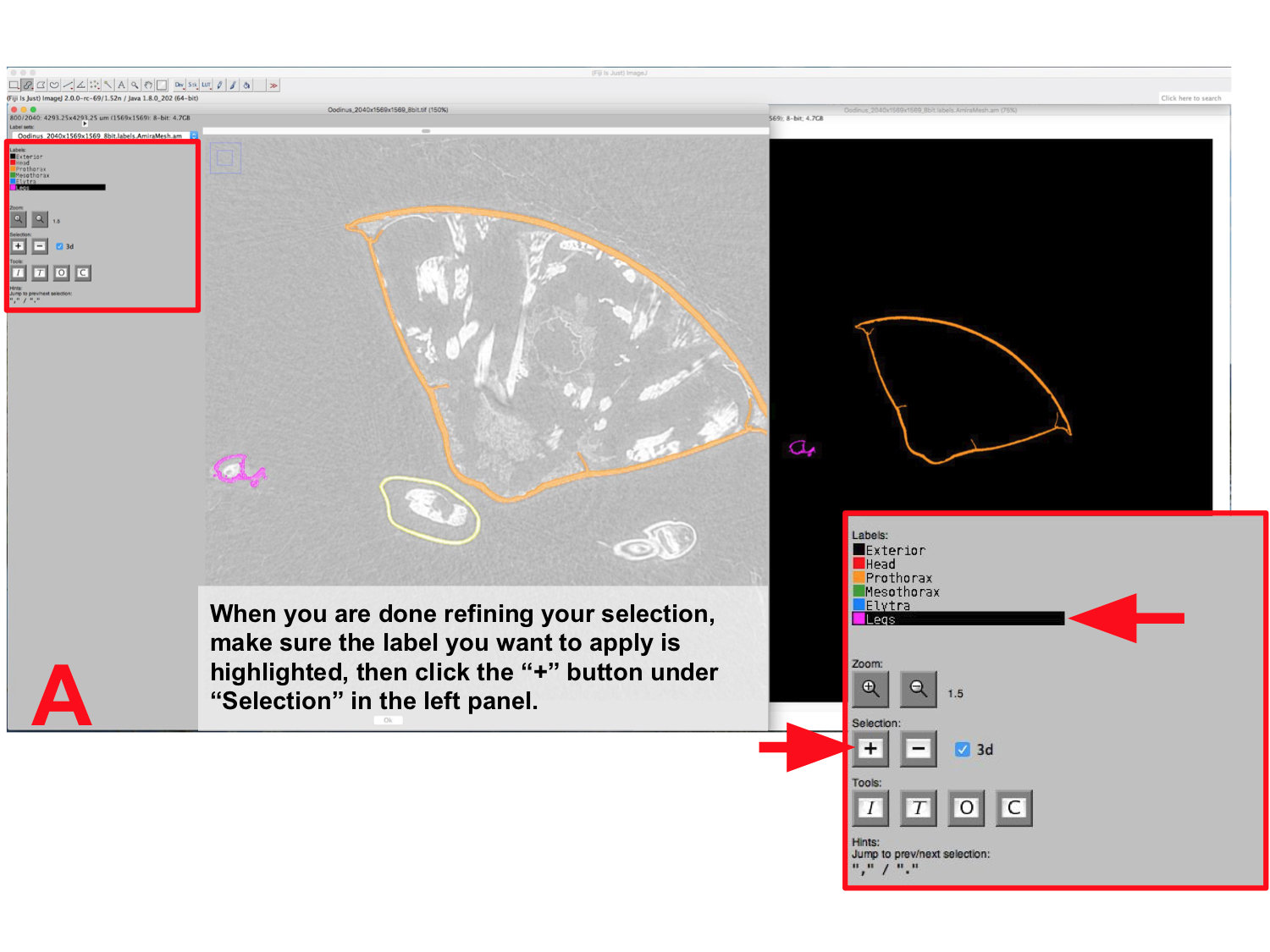


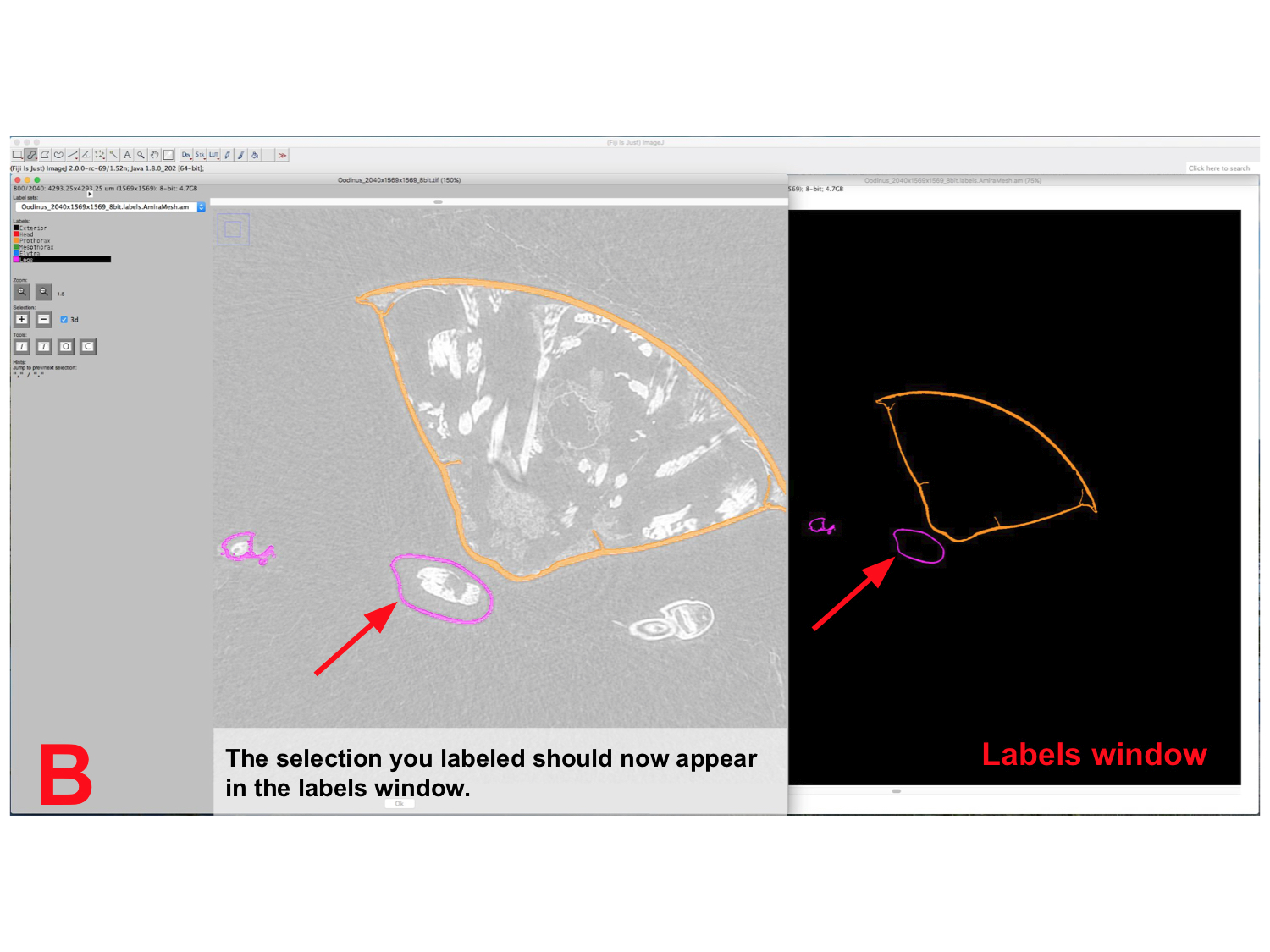


**Figure S1-3:** Adding a selection to a label.
