# Supplementary material for: The Natural Historian's Guide to the CT Galaxy: Step-by-Step Instructions for Preparing and Analyzing Computed Tomographic (CT) Data Using Cross-Platform, Open Access Software
Source: Integr Org Biol. 2020 Apr 10;2(1):obaa009. doi: 10.1093/iob/obaa009 (PMC7671151; doi:10.1093/iob/obaa009)
Supplement: obaa009_Supplementary_Data [file obaa009_supplementary_data.zip › Supplementary Data 2 Step 9d_revision_191126.docx]

**SUPPLEMENTARY DATA 2 STEP 9D: Simple tricks and nonsense: volume rendering for meristic counts & natural history insights**

Volume rendering is not just useful for producing vast quantities of high-quality anatomical schematics for osteology and taxonomy in general (e.g. Conway et al., 2017, 2018). The speed with which low-quality volume renders can be generated is also useful for data collection, either for meristics and observational data, or for quantifying simple metrics like percent coverage (in combination with image software like Fiji). For example, after volume rendering specimens, we have been able to quickly and easily count skeletal elements like: (1) abdominal serrae or supraneural spines in piranhas (a useful taxonomic character), (2) evidence of damage or healing to parts of the skeleton in characoids and catfishes (Kolmann et al., 2018b), and even (3) stomach contents (Kolmann et al., 2018a). With some ingenuity, static CT scans can also elucidate some questions about skeletal motion and conformational changes in shape, for example, Kolmann et al. (2016) used sequential CT scanning to capture how tooth orientation changes during jaw protrusion in stingrays: radio-transparent spray insulation foam was used to ‘pose’ specimens with their jaws retracted and protruded.

Another simple volume-rendering method for collecting meristic data is Maximum Intensity Projection (MIP). This tool is usually an extension of volume rendering functions, available in most CT software packages, that finds the brightest pixels (i.e. those with the highest attenuation values) and preferentially displays these values relative to considerably less dense tissues (e.g. Figure SD2-1, below). This method starkly contrasts denser vs. softer tissues and is used in the medical community to pinpoint malignant growths. In biology, this method has been used to image otoliths, hyperostoses, and *in situ* tooth replacement for example (Paig-Tran et al., 2016; Kolmann et al., 2019).

**Figure S2-1:** Examples of volume rendering (A) and maximum intensity projection (B) and their utility in quickly assessing the morphology of anatomical structures.
