# Supplementary material for: The Natural Historian's Guide to the CT Galaxy: Step-by-Step Instructions for Preparing and Analyzing Computed Tomographic (CT) Data Using Cross-Platform, Open Access Software
Source: Integr Org Biol. 2020 Apr 10;2(1):obaa009. doi: 10.1093/iob/obaa009 (PMC7671151; doi:10.1093/iob/obaa009)
Supplement: obaa009_Supplementary_Data [file obaa009_supplementary_data.zip › Supplementary Script 1_200307.rtf]

//-----------------------------------------------------------------------------//// This script was written by Dr. Russell Garwood using modifications// of an example macro from the ImageJ website. Questions regarding// the script should be directed to russell.garwood@gmail.com / // google russell garwood.// //----------------------------------------------a-------------------------------////------------------------ Process Virtual Stack ------------------------------////// This macro process the images in a virtual stack.//// It allows you to change brightness and contrast on CT slices and will then// save the processed images in a folder of your choice.//// If you want to crop the data, just draw a box on and it'll save the cropped// region.////---------------------------- To import data ---------------------------------//////---- DICOM/TIFF stack:// 1) The virtualstack can be opened using File->Import->Image Sequence if you have// a DICOM/TIFF stack.////---- For VGI/VOL:// 1) File->Import->Raw// 2) Set Image type to "32-bit Signed" // 3) Width height and image number: can be found in the VGI, in that order after// "size = "// 4) Untick: "White is Zero"// 5) Tick: "Little-endian byte order"// 6) Untick: "Open all files in folder"// 7) Tick: "Use virtual stack"////-------------------- To alter brightness and contrast -----------------------////// The contrast may be way off on import, and you'll probably want to alter it// anyway before saving for this. Use "Image->Adjust->Brightness/Contrast.// When finished add these figures to the variables below, and save this file.// eg.//  minValue = 1113919104.00;//  maxValue = 1127656320.00;minValue = ;maxValue = ;////-------------------------------- Output Format ------------------------------//// Change format here to bmp, png, or tiff if needed:outputFormat = "bmp";////-------------------------------- To run macro -------------------------------//// To run macro: plugins->macro->run->choose contrast and save.txt////-----------------------------------------------------------------------------////-------------------------------- Macro --------------------------------------//if (nSlices==1) exit("Stack required");dir = getDirectory("Choose destination directory for BMP stack");setBatchMode(true);id = getImageID;for (i=1; i<= nSlices; i++) {    showProgress(i, nSlices);    selectImage(id);    setSlice(i);    name = getMetadata;    run("Duplicate...", "title=temp");run("Brightness/Contrast...");setMinAndMax(minValue, maxValue);if(i<=10) saveAs(outputFormat, dir+"000"+(i-1));       if(i>10&&i<=100) saveAs(outputFormat, dir+"00"+(i-1));       if(i>100&&i<=1000) saveAs(outputFormat, dir+"0"+(i-1));if(i>1000) saveAs(outputFormat, dir+(i-1));close();}setBatchMode(false);//-----------------------------------------------------------------------------//
